# Graphics processing units in bioinformatics, computational biology and systems biology

**DOI:** 10.1093/bib/bbw058

**Published:** 2016-07-07

**Authors:** Marco S Nobile, Paolo Cazzaniga, Andrea Tangherloni, Daniela Besozzi

**Affiliations:** 1 Department of Informatics, Systems and Communication, University of Milano-Bicocca, Milano, Italy; 2 Department of Human and Social Sciences, University of Bergamo, Bergamo, Italy; 3 SYSBIO.IT Centre of Systems Biology, Milano, Italy

**Keywords:** graphics processing units, CUDA, high-performance computing, bioinformatics, computational biology, systems biology

## Abstract

Several studies in Bioinformatics, Computational Biology and Systems Biology rely on the definition of physico-chemical or mathematical models of biological systems at different scales and levels of complexity, ranging from the interaction of atoms in single molecules up to genome-wide interaction networks. Traditional computational methods and software tools developed in these research fields share a common trait: they can be computationally demanding on Central Processing Units (CPUs), therefore limiting their applicability in many circumstances. To overcome this issue, general-purpose Graphics Processing Units (GPUs) are gaining an increasing attention by the scientific community, as they can considerably reduce the running time required by standard CPU-based software, and allow more intensive investigations of biological systems. In this review, we present a collection of GPU tools recently developed to perform computational analyses in life science disciplines, emphasizing the advantages and the drawbacks in the use of these parallel architectures. The complete list of GPU-powered tools here reviewed is available at http://bit.ly/gputools.

## Introduction

Typical applications in Bioinformatics, Computational Biology and Systems Biology exploit either physico-chemical or mathematical modeling, characterized by different scales of granularity, abstraction levels and goals, which are chosen according to the nature of the biological system under investigation—from single molecular structures up to genome-wide networks—and to the purpose of the modeling itself.

Molecular dynamics, for instance, simulates the physical movements of atoms in biomolecules by calculating the forces acting on each atom, considering bonded or non-bonded interactions [1, 2]. Sequence alignment methods scale the abstraction level from atoms to RNA or DNA molecules, and then up to whole genomes, to the aim of combining or interpreting nucleotide sequences by means of string-based algorithms [3]. Systems Biology considers instead the emergent properties of complex biological systems—up to whole cells and organs [4, 5]—focusing either on topological properties or flux distributions of large-scale networks, or on the dynamical behavior of their molecular components (e.g. genes, proteins, metabolites).

Although these disciplines are characterized by different goals, deal with systems at different scales of complexity and require completely different computational methodologies, they share an ideal *trait d’union*: all of them are computationally challenging [6–8]. Computers based on Central Processing Units (CPUs) are constantly improving, offering improved performances thanks to the parallelism granted by multi-threading and the vector instructions provided by e.g. Streaming SIMD Extensions (SSE) [9]. Still, computational analyses in life science disciplines often lie on the boundary of feasibility because of the huge computational costs they require on CPUs. Hence, an intense research is focusing on the optimization of algorithms and data structures in these fields; anyway, many computational methods can already benefit from non-conventional computing architectures. In particular, parallel infrastructures can be used to strongly reduce the prohibitive running times of these methods, by distributing the workload over multiple independent computing units. It is worth noting, however, that not all problems can be parallelized, as they are inherently sequential.

In the context of high-performance computing (HPC), the traditional solutions for distributed architectures are represented by computer clusters and grid computing [10, 11]. Although these infrastructures are characterized by some considerable drawbacks, in general they are largely used by the scientific community because they allow to execute the available computational methods with minimal changes to the existing CPU code. A third way to distributed computation is the emergent field of cloud computing, whereby private companies offer a pool of computation resources (e.g. computers, storage) attainable on demand and ubiquitously over the Internet. Cloud computing mitigates some problems of classic distributed architectures; however, it is affected by the fact that data are stored on servers owned by private companies, bringing about issues of privacy, potential piracy, continuity of the service, ‘data lock-in’, along with typical problems of Big Data e.g. transferring terabyte-scale data to and from the cloud [12]. An alternative option for HPC consists in the use of reconfigurable hardware platforms such as Field Programmable Gates Arrays (FPGAs) [13], which require dedicated hardware and specific programming skills for circuits design.

In the latter years, a completely different approach to HPC gained ground: the use of general-purpose multi-core devices like Many Integrated Cores (MIC) co-processors and Graphics Processing Units (GPUs). In particular, GPUs are gaining popularity, as they are pervasive, relatively cheap and extremely efficient parallel multi-core co-processors, giving access to low-cost, energy-efficient means to achieve tera-scale performances on common workstations (and peta-scale performances on GPU-equipped supercomputers [14, 15]). However, tera-scale performances represent a theoretical peak that can be achieved only by distributing the whole workload across all available cores [16] and by leveraging the high-performance memories on the GPU, two circumstances that are seldom simultaneously verified. Even in sub-optimal conditions, though, GPUs can achieve the same performances of other HPC infrastructures, albeit with a single machine and, remarkably, without the need for job scheduling or the transfer of confidential information. Being GPU’s one of the most efficient and largely exploited parallel technology, in this article we provide a review of recent GPU-based tools for biological applications, discussing both their strengths and limitations. Indeed, despite its relevant performance, also general-purpose GPU (GPGPU) computing has some drawbacks. The first is related to the fact that GPUs are mainly designed to provide the ‘Same Instruction Multiple Data’ (SIMD) parallelism, that is, all cores in the GPU are supposed to execute the same instructions on different input data (For the sake of completeness, we report that, on the most recent architectures, concurrent kernels can be executed on a single GPU, providing a hybrid SIMD-MIMD execution. Additional information about concurrent kernels is provided in Supplementary File 2). This is radically different from the ‘Multiple Instruction Multiple Data’ (MIMD) paradigm of computer clusters and grid computing, whereby all computing units are independent, asynchronous, can work on different data and execute different code. As SIMD is not the usual execution strategy for existing CPU implementations, the CPU code cannot be directly ported to the GPU’s architecture. In general, the CPU code needs to be rewritten for GPUs, which are completely different architectures and support a different set of functionalities, as well as different libraries. In addition, the complex hierarchy of memories and the limited amount of high-performance memories available on GPUs generally require a redesign of the existing algorithms, to better fit and fully leverage this architecture. Thus, from the point of view of the software developer, GPU programming still remains a challenging task [17]. Table 1 presents an overview of various HPC infrastructures, together with their architectural features, advantages and limits.

**Table 1:** High-performance computing architectures: advantages and drawbacks

HPC type	Architecture	Advantages	Drawbacks	Computing paradigm
Computer cluster	Set of interconnected computers controlled by a centralized scheduler	Require minimal changes to the existing source code of CPU programs, with the exception of possible modifications necessary for message passing	Expensive, characterized by relevant energy consumption and requires maintenance	MIMD
Grid computing	Set of geographically distributed and logically organized (heterogeneous) computing resources	Require minimal changes to the existing source code of CPU programs, with the exception of possible modifications necessary for message passing	Generally based on ‘volunteering’: computer owners donate resources (e.g. computing power, storage) to a specific project; no guarantee about the availability of remote computers: some allocated tasks could never be processed and need to be reassigned; remote computers might not be completely trustworthy	MIMD
Cloud computing	Pool of computation resources (e.g. computers, storage) offered by private companies, attainable on demand and ubiquitously over the Internet	Mitigate some problems like the costs of the infrastructure and its maintenance	Data are stored on servers owned by private companies; issues of privacy, potential piracy, espionage, international legal conflicts, continuity of the service (e.g. owing to some malfunctioning, DDoS attacks, or Internet connection problems)	MIMD
GPU	Dedicated parallel co-processor, formerly devoted to real-time rendering of computer graphics, nowadays present in every common computer	High number of programmable computing units allow the execution of thousands simultaneous threads. Availability of high-performance local memories	Based on a modified SIMD computing paradigm: conditional branches imply serialization of threads’ execution. GPU’s peculiar architecture generally requires code rewriting and algorithms redesign	SIMD (although temporary divergence is allowed)
MIC	Dedicated parallel co-processor installable in common desktop computers, workstations and servers	Similar to GPUs but based on the conventional ×86 instructions set: existing CPU code, in principle, might be ported without any modification. All cores are independent	Fewer cores with respect to latest GPUs. To achieve GPU-like performances, modification of existing CPU code to exploit vector instructions are required	MIMD
FPGA	Integrated circuits containing an array of programmable logic blocks	Able to implement a digital circuit, which directly performs purpose-specific tasks (unlike general-purpose software tools). Such tasks are executed on a dedicated hardware without any computational overhead (e.g. those related to the operating system)	Generally programmed using a descriptive language (e.g. VHDL, Verilog [18]), which can be cumbersome. Debugging using digital circuits simulators might be complicated and not realistic. Experience with circuit design optimization might be necessary to execute tasks using the highest clock frequency	Dedicated hardware

In the context of GPGPU computing, Nvidia’s CUDA (Compute Unified Device Architecture) is the most used library for the development of GPU-based tools in the fields of Bioinformatics, Computational Biology and Systems Biology, representing the *standard de facto* for scientific computation. CUDA can only exploit Nvidia GPUs, but alternative solutions exist, such as Microsoft DirectCompute (which can be used only with Microsoft’s Windows operating system) and the platform-independent library OpenCL (which can also leverage AMD/ATI GPUs). In this review we focus on available GPU-powered tools, mainly based on CUDA, for computational analyses in life-science fields. In particular, we present recent GPU-accelerated methodologies developed for sequence alignment, molecular dynamics, molecular docking, prediction and searching of molecular structures, simulation of the temporal dynamics of cellular processes and analysis methods in Systems Biology. Owing to space limits, a collection of additional applications of GPUs developed to deal with other life-science problems—spectral analysis, genome-wide analysis, Bayesian inference, movement tracking, quantum chemistry—is provided in Supplementary File 1. The complete list of the GPU-powered tools presented in this review is also available at http://bit.ly/gputools. Developers of GPU-based tools for the aforementioned disciplines are invited to contact the authors to add their software to the webpage.

This review is structured in a way that each section can be read independently from the others, so that the reader can freely skip topics not related to his/her own interests, without compromising the comprehension of the overall contents. The works presented in this review were chosen by taking into account their chronological appearance, preferring the most recent implementations over earlier tools, some of which were previously reviewed elsewhere [19–21]. Among the cited works, we identified, when possible, the most performing tool for each specific task, and report the computational speed-up claimed by the authors. Except where stated otherwise, all tools are assumed to be implemented using the C/C ++ language.

The review is mainly conceived for end users of computational tools in Bioinformatics, Computational Biology and Systems Biology—independently of their educational background or research expertise—who can be well-acquainted with available CPU-based software in these fields, but might profitably find out how GPUs can give a boost to their analyses and research outcomes. In particular, end users with a main biological background can take advantage of this review to get a widespread overview of existing GPU-powered tools, and leverage them to reduce the running times of routine computational analysis. On the other hand, end users with a main Bioinformatics or Computer Science background, but having no expertise in GPU programming, can take the opportunity to learn the main pitfalls as well as some useful strategies to fully leverage the potentiality of GPUs. In general, readers not familiar with GPUs and CUDA, but interested in better understanding the implementation issues discussed hereby, can find in Supplementary File 2 a detailed description of the main concepts related to this HPC solution (e.g. thread, block, grid, memory hierarchy, streaming multiprocessor, warp voting, coalesced patterns). The aim of Supplementary File 2 is to make this review self-contained with respect to all GPU-related issues that are either mentioned or discussed in what follows. Finally, Supplementary File 3 provides more technical details (e.g. peak processing power, global memory size, power consumption) about the Nvidia GPUs that have been used in the papers cited in this review.

This work ends with a discussion about future trends of GPU-powered analysis of biological systems. We stress the fact that, except when authors of reviewed papers performed themselves a direct comparison between various GPU-powered tools, the architectural differences of the workstations used for their tests prevented us from performing a fair comparison among all different implementations. As a consequence, we shall not provide here a ranking of the different tools according to their computational performance. Indeed, such a ranking would require the re-implementation and testing of all algorithms by using the same hardware as well as different problem instances, which is far beyond the scope of this review.

## Sequence alignment

The use of parallel co-processors has proven to be beneficial for genomic sequence analysis (Table 2). In this context, the advantages achievable with GPU-powered tools is of particular importance when considering next-generation sequencing (NGS) methodologies, which allow to parallelize the sequencing process by producing a huge number of subsequences (named ‘short reads’) of the target genome, which must be realigned against a reference sequence. Therefore, in the case of high-throughput NGS methods, a typical run produces billions of reads, making the alignment problem a challenging computational task, possibly requiring long running times on CPUs.

**Table 2:** GPU-powered tools for sequence alignment, along with the speed-up achieved and the solutions used for code parallelization

Sequence alignment				
	Tool name	Speed-up	Parallel solution	Reference
Sequence alignment based on BWT	BarraCUDA	–	GPU	[22]
Sequence alignment based on BWT	CUSHAWGPU	–	GPU	[23]
Sequence alignment based on BWT	GPU-BWT	–	GPU	[24]
Sequence alignment based on BWT	SOAP3	–	CPU-GPU	[25]
Sequence alignment based on hash table	SARUMAN	–	GPU	[26]
Sequence alignment with gaps based on BWT	SOAP3-dp	–	CPU-GPU	[27]
Tool to map SNP exploiting SOAP3-dp	G-SNPM	–	CPU-GPU	[28]
Sequence alignment exploiting SOAP3-dp	G-CNV	18×	CPU-GPU	[29]
Alignment of gapped short reads with Bowtie2 algorithm	nvBowtie	8×	GPU	[30]
Alignment of gapped short reads with Bowtie2 algorithm	MaxSSmap	–	GPU	[31]
Reads assembly exploiting the de Bruijn approach	GPU-Euler	5×	GPU	[32]
Reads assembly exploiting the de Bruijn approach	MEGAHIT	2×	GPU	[33]
Sequence alignment (against database) tool	–	2×	GPU	[34]
Sequence alignment (against database) tool	CUDA-BLASTP	6×	GPU	[35]
Sequence alignment (against database) tool	G-BLASTN	14.8×	GPU	[36]
Sequence alignment with Smith-Waterman method	SW#	–	GPU	[37]
Sequence alignment based on suffix tree	MUMmerGPU 2.0	4×	GPU	[38]
Sequence similarity detection	GPU CAST	10×	GPU	[39]
Sequence similarity detection based on profiled Hidden Markov Models	CUDAMPF	11–37×	GPU	[40]
Multiple sequence alignment with Clustal	CUDAClustal	2×	GPU	[41]
Multiple sequence alignment with Clustal	GPU-REMuSiC	–	GPU	[42]

Regardless of the used sequencing methodology, existing aligners can be roughly partitioned into two classes, according to the data structure they exploit: hash tables and suffix/prefix trees. The latter approach requires particular algorithms and data structures like the Burrows-Wheeler Transform (BWT) [43] and the FM-index [44]. In this context, multiple tools based on CUDA have already been developed: BarraCUDA [22], CUSHAW [23], GPU-BWT [24] and SOAP3 [25] (all based on BWT), and SARUMAN [26] (based on hashing).

SOAP3 is based on a modified version of BWT tailored for GPU execution—named GPU-2BWT—which was redesigned to reduce the accesses to the global memory; the access time to the memory was further optimized by using coalesced access patterns. Moreover, SOAP3 performs a pre-processing of sequences to identify those patterns—named ‘hard patterns’—that would cause a high level of branching in CUDA kernels: hard patterns are processed separately, thus reducing the serialization of threads execution. SOAP3 is also able to perform heterogeneous computation, by simultaneously leveraging both CPU and GPU. In 2013, a special version of SOAP3, named SOAP3-dp [27], able to cope with gapped alignment and implementing a memory-optimized dynamic programming methodology, was proposed and compared against CUSHAW and BarraCUDA. According to this comparison on both real and synthetic data, SOAP3-dp turned out to be the fastest implementation to date, outperforming the other methodologies, also from the point of view of the sensitivity. SOAP3-dp represents the foundation of G-SNPM [28], another GPU-based tool for mapping single nucleotide polymorphisms (SNP) on a reference genome. Moreover, SOAP3-dp is also exploited by G-CNV [29], a GPU-powered tool that accelerates the preparatory operations necessary for copy number variations detection (e.g. low-quality sequences filtering, low-quality nucleotides masking, removal of duplicate reads and ambiguous mappings). Thanks to GPU acceleration, G-CNV offers up to 18× acceleration with respect to state-of-the-art methods.

At the beginning of 2015, Nvidia published the first official release of its NVBIO [45] library, which gives access to a variety of data structures and algorithms useful for sequence alignment (e.g. packed strings, FM-index, BWT, dynamic programming alignment), providing procedures for transparent decompression and processing of the most widespread input formats (e.g. FASTA, FASTQ, BAM). Built on top of the NVBIO library, nvBowtie is a GPU-accelerated re-engineering of the Bowtie2 algorithm [30] for the alignment of gapped short reads. According to Nvidia, nvBowtie allows an 8× speed-up with respect to the highly optimized CPU-bound version. In addition to this, MaxSSmap [31] was proposed as a further GPU-powered tool for mapping short reads with gapped alignment, designed to attain a higher level of accuracy with respect to competitors.

When the reference genome is not available, the problem becomes to re-assembly *de novo* a target genome from the reads. Two GPU-based software tools are available for reads assembly: GPU-Euler [32] and MEGAHIT [33], both exploiting a de Bruijn approach, whereby the overlaps between input reads are identified and used to create a graph of contiguous sequences. Then, the Eulerian path over this graph represents the re-assembled genome. The speed-up of GPU-Euler is about 5× with respect to the sequential version, using a Nvidia QUADRO FX 5800. According to the authors, GPU-Euler's reduced speed-up is owing to memory optimization: none of the high-performance memories (e.g. shared memory, texture memory) were exploited in the current implementation, although they could reduce the latencies owing to the hash table look-up. MEGAHIT, instead, halves the running time of the re-assembly with respect to a sequential execution. Unfortunately, the performances of the two algorithms had never been compared.

As in the case of the problem of short reads alignment against a reference genome, the alignment of primary sequences consists in a query sequence that is compared with a library of sequences, to identify ‘similar’ ones. The most widespread algorithm to tackle this problem is the BLAST heuristic [46, 47]. The first attempts in accelerating BLAST on GPUs [34, 35] were outperformed by G-BLASTN [36], which offers a 14.8× speed-up and guarantees identical results to traditional BLAST. An alternative algorithm for sequence alignment is the Smith-Waterman [48] dynamic programming method, which is usually impracticable for long DNA sequences owing to its quadratic time and space computational complexity. Thanks to advanced space optimization and the adoption of GPU acceleration, SW# [37] offers genome-wide alignments based on Smith-Waterman with a speed-up of two orders of magnitude with respect to equivalent CPU implementations, using a Nvidia GeForce GTX 570. Smith-Waterman is also the basis of CUDA-SW ++3 [49], used to provide protein sequence search, based on pairwise alignment. This tool—which is the result of a long series of optimizations, outperforming all previous solutions [50–52]—represents a heterogeneous implementation able to carry out concurrent CPU and GPU executions. Both architectures are intensively exploited to maximize the speed-up: on the one hand, CUDA-SW ++3 leverages SSE vector extensions and multi-threading on the CPU; on the other hand, it exploits PTX SIMD instructions (i.e. vector assembly code) to further increase the level of parallelism (see Supplementary File 2). According to the authors, CUDA-SW ++3 running on a GTX690 is up to 3.2× faster than CUDA-SW ++2; it is also 5× faster than SWIPE [53] and 11× faster than BLAST+ [54], both running in multi-threaded fashion on an Intel i7 2700K 3.5 GHz CPU.

MUMmer uses an alternative approach, based on a suffix tree, requiring linear space and enabling substring matching in linear time [55]. Thanks to GPU acceleration and a careful data layout optimization, MUMmerGPU 2.0 [38] provides a 4× speed-up with respect to classic MUMmer.

The problem of sequence similarity is also tackled by GPU_CAST [39], a parallel version of the CAST software [56] ported to CUDA. CAST performs optimized local sequence similarities by detecting the ‘low-complexity regions’ (LCR), i.e. biologically unrelated sequences owing to compositionally biased sequence pairs. By masking LCR, CAST significantly improves the reliability of homology detection. Thanks to GPU acceleration, GPU_CAST allows a speed-up ranging from 5× up to 10× with respect to the classic multi-threaded version, with a relevant part of the execution time (30% on average) owing to memory transfers.

The problem of sequence similarity, for the detection of common motifs, is tackled by the HMMER3 pipeline, which is based on profiled Hidden Markov Models [57]. HMMER3 is a strongly optimized tool, fully leveraging CPU’s multi-threading and vector instructions. Hence, repeated parallelization attempts did not lead to a significant speed-up, except in the case of CUDAMPF [40], a careful implementation, which leverages multiple recent CUDA features (at the time of writing) like vector instructions, real-time compilation for loop unrolling and dynamic kernel switching according to task workloads. The reported speed-up of CUDAMPF ranges between 11× and 37× with respect to an optimized CPU version of HMMER3, while the GPU implementation of HMMER presented by Ganesan *et al.* [58] does not achieve any relevant speed-up.

The last problem we consider is the alignment of multiple sequences (MSA) for the identification of similar residues. This problem could be tackled by means of dynamic programming, but this strategy is generally unfeasible because of its exponential space computational complexity [41]. An alternative approach to MSA is the progressive three-stage alignment performed by Clustal [59]: (i) pair-wise alignment of all sequences; (ii) construction of the phylogenetic tree; (iii) use of the phylogenetic tree to perform the multiple alignments. The GPU-accelerated version CUDAClustal [41] globally improved the performances by 2× using a GeForce GTX 295, although the parallelization of the first stage—implemented by means of strip-wise parallel calculation of the similarity matrices—allows a 30× speed-up with respect to the sequential version. In a similar vein, GPU-REMuSiC [42] performs GPU-accelerated progressive MSA. However, differently from CUDAClustal, this tool allows to specify regular expressions to apply constraints during the final alignment phase. According to [42], the speed-up of GPU-REMuSiC is relevant, especially because it is natively able to distribute the calculations across multiple GPUs.

## Molecular dynamics

The physical movements of macromolecules, such as proteins, can be simulated by means of molecular mechanics methods. This computational analysis is highly significant, as large-scale conformational rearrangements are involved in signal transduction, enzyme catalysis and protein folding [60].

Molecular dynamics [2] describes the movements of molecules in space by numerically solving Newton’s laws of motion, i.e. by calculating the force, position and velocity of each atom over a series of time steps. Molecular dynamics is computationally challenging: the length of the time step of a simulation is generally limited to <5 fs, while the overall time of the phenomenon is, typically, in the order of ns or s. Molecular dynamics methods have been improved over the years, starting from the first 10 ps-long simulation of a molecule consisting of 500 atoms [61], passing through experiments where the movement of small enzymes was simulated on a s time scale [62], up to proteins composed of millions of atoms [63]. Being computationally intensive, many implementations of molecular dynamics algorithms started to exploit CPU-based large-scale supercomputers [64, 65]. The main limitations of these solutions regard the high costs of supercomputers, the necessity of implementing a scheduler to handle the parallel execution of the code and the maintenance issues (see Table 1).

Nowadays, there exist different molecular dynamics simulators, implemented by means of CUDA, that completely rely on GPUs (Table 3). Molecular dynamics can be parallelized at the level of atoms, or considering either the interactions among atoms or some spatial partitioning of the molecules [73]. For instance, a new algorithm for non-bonded short-range interactions within the atoms system was introduced by Liu *et al.* [66]. Tested on protein systems with up to 131 072 atoms, it achieved a 11× speed-up exploiting a Nvidia GeForce 8800 GTX compared with an optimized code exploiting the SSE instruction set on a Pentium IV 3.0 GHz. A CUDA implementation of generalized explicit solvent all-atom classic molecular dynamics within the AMBER package was introduced in [67]. The feasibility of different GPUs for molecular dynamics simulations was evaluated considering the maximum number of atoms that video cards could handle, according to the available memory. Then, performance tests were conducted on protein systems with up to 408 576 atoms; the achieved speed-up was 2–5× comparing the execution on different GPUs (i.e. GTX 580, M2090, K10, GTX 680, K20X, GTX TITAN), with respect to the parallel CPU-based implementation using up to 384 Intel Sandy Bridge E5-2670 2.6 GHz.

**Table 3:** GPU-powered tools for molecular dynamics, along with the speed-up achieved and the solutions used for code parallelization

Molecular dynamics				
	Tool name	Speed-up	Parallel solution	Reference
Non-bonded short-range interactions	–	11×	GPU	[66]
Explicit solvent using the particle mesh Ewald scheme for the long-range electrostatic interactions	–	2–5×	GPU	[67]
Non-Ewald scheme for long-range electrostatic interactions	–	100×	multi-GPU	[68]
Standard covalent and non-covalent interactions with implicit solvent	OpenMM	–	GPU	[69]
Non-bonded and bonded interactions, charge equilibration procedure	PuReMD	16×	GPU	[70]
Energy conservation for explicit solvent models	MOIL-opt	10×	CPU-GPU	[71]
Electrostatics and generalized Born implicit solvent model	LTMD	5.8×	CPU-GPU	[72]

Mashimo *et al.* [68] presented a CUDA-based implementation of non-Ewald scheme for long-range electrostatic interactions, whose performances were assessed by simulating protein systems with a number of atoms ranging from 38 453 to 1 004 847. This implementation consists in a MPI/GPU-combined parallel program, whose execution on a rack equipped with 4 Nvidia M2090 achieved a 100× speed-up with respect to the sequential counterpart executed on a CPU Intel E5 2.6 GHz. Finally, OpenMM [69] is an open-source software for molecular dynamics simulation for different HPC architectures (it supports GPUs with both CUDA and OpenCL frameworks). OpenMM was tested on a benchmark model with 23 558 atoms, allowing the simulation of tens of ns/day with a Nvidia GTX 580 and a Nvidia C2070 (no quantitative results about the speed-up with respect to CPU-based applications were given).

We highlight that, when implementing molecular dynamics methods on GPUs, some general issues should be taken into account. First, GPUs are not suitable for the parallelization of every kind of task. Some attempts tried to implement the entire molecular dynamics code with CUDA, resulting in a lack of performance, caused by frequent access to high-latency memories or by functions requiring more demanding double precision accuracy (to this aim, some work focused on the definition of ‘precision’ methods to avoid the necessity of double-precision arithmetic on the GPU [74]). Other approaches exploited GPUs to generate random numbers required by specific problems of Dissipative Particle Dynamics (an extension of molecular dynamics), achieving a 2–7× speed-up with respect to CPUs [75]. Second, the optimal number of threads per block should be carefully evaluated considering the application [76], as well as the number of threads per atom that should be launched according to the kernel, to the aim of increasing the speed-up (see, for instance, the GPU implementation of PuReMD [70]). Third, the load between CPU and GPU should be balanced so that both devices would spend the same amount of time on their assigned task. However, this is challenging and not every molecular dynamics implementation that exploits both CPU and GPU is able to fulfill this requirement. Fourth, different languages (e.g. CUDA, C, C ++, Fortran) are typically used when developing code, resulting in a hardware-specific source code, usually hard to maintain. In these cases, minor changes in the operating system, compiler version or hardware could lead to dramatic source code and compilation changes, possibly impairing the usability of the application.

Having this in mind, different kinds of molecular dynamics methods rely on hybrid implementations that exploit both CPUs and GPUs. For instance, a hybrid CPU-GPU implementation with CUDA of MOIL (i.e. energy-conserving molecular dynamics) was proposed in [71]. This implementation was tested by using a quad-core AMD Phenom II X4 965 3.4 GHz coupled with a Nvidia GTX 480, for the simulation of molecular systems with up to 23 536 atoms, and it achieved a 10× speed-up with respect to a strictly CPU-bound multi-threaded counterpart. As a final example, a long time step molecular dynamics with hybrid CPU-GPU implementation was described by Sweet *et al.* [72]. In this work, GPUs accelerate the computation of electrostatics and generalized Born implicit solvent model, while the CPU handles both the remaining part of the computation and the communications. The performance of this method was tested on molecular systems with up to 1251 atoms, achieving a 5.8× speed-up with respect to implementations entirely based on the GPU.

We refer the interested reader to the review presented by Loukatou *et al.* [77] for a further list of GPU-based software for molecular dynamics. 

## Molecular docking

The aim of molecular docking is to identify the best ‘lock-and-key’ matching between two molecules, e.g. protein–protein, protein–ligand or protein–DNA complex [78]. This method represents indeed a fundamental approach for drug design [79]. Computational approaches for molecular docking usually assume that the molecules are rigid, semi-flexible or flexible; in any case, the major challenge concerns the sampling of the conformational space, a task that is time-consuming. In its general formulation, no additional data other than the atomic coordinates of the molecules are used; however, further biochemical information can be considered (e.g. the binding sites of the molecules).

One of the first attempts in accelerating molecular docking on GPUs was introduced by Ritchie and Venkatraman [80], who presented an implementation of the *Hex* spherical polar Fourier protein docking algorithm to identify the initial rigid body stage of the protein–protein interaction. The Fast Fourier transform (FFT) represents the main GPU-accelerated part of the implementation, and relies on the cuFFT library [81] (see also Supplementary File 2). FFT is calculated by means of a *divide et impera* algorithm, which is perfectly suitable to distribute calculations over GPU’s multiple threads. Because of that, results showed a 45× speed-up on a Nvidia GeForce GTX 285 with respect to the CPU, reducing to the order of seconds the time required for protein docking calculations.

A different GPU-powered strategy for conformation generation and scoring functions was presented by Korb *et al.* [82]. Considering protein–protein and protein–ligand systems (with rigid protein and flexible ligand), the authors achieved a 50× and a 16× speed-up, respectively, by using a Nvidia GeForce 8800 GTX with respect to a highly optimized CPU implementation. The main bottleneck of this work concerns the performance of the parallel ant colony optimization algorithm to identify the best conformation that, compared with the CPU-based counterpart, requires a higher number of scoring function evaluations to reach a comparable average success rate.

Simosen *et al.* [83] presented a GPU implementation of MolDock, a method for performing high-accuracy flexible molecular docking, focused on protein–ligand complexes to search drug candidates. This method exploits a variant of differential evolution to efficiently explore the search space of the candidate binding modes (i.e. the possible interactions between ligands and a protein). This implementation achieved a speed-up of 27.4× by using a Nvidia GeForce 8800 GT, with respect to the CPU counterpart. Authors also implemented a multi-threaded version of MolDock, which achieved a 3.3× speed-up on a 4 cores Intel Core 2 with respect to the single-threaded CPU implementation. According to this result, the speed-up of the GPU implementation is roughly reduced to about 8× if compared with the multi-threaded version of MolDock.

More recent applications for molecular docking are ppsAlign [84], the protein–DNA method proposed by Wu *et al.* [85] and MEGADOCK [86]. ppsAlign is a method for large-scale protein structure alignment, which exploits the parallelism provided by GPU for the sequence alignment steps required for structure comparison. This method was tested on a Nvidia Tesla C2050, achieving up to 39× speed-up with respect to other state-of-the-art CPU-based methods. The protein–DNA method is a semi-flexible molecular docking approach implemented on the GPU, which integrates Monte Carlo simulation with simulated annealing [87] to accelerate and improve docking quality. The single GPU version achieved a 28× speed-up by using a Nvidia M2070 with respect to the single CPU counterpart; other tests on a cluster of GPUs highlighted that the computational power of 128 GPUs is comparable with that of 3600 CPU cores.

MEGADOCK is an approach for rigid protein–protein interactions implementing the Katchalski-Katzir algorithm with the traditional Fast Fourier transform rigid-docking scheme, accelerated on supercomputers equipped with GPUs (in particular, MEGADOCK was implemented for single GPU, multi-GPUs and CPU). The computational experiments were performed on the TSUBAME 2.5 supercomputer—having each node equipped with 3 Nvidia Tesla K20X—considering 30 976 protein pairs of a cross-docking study between 176 receptors and 176 ligands. The claimed speed-up reduces the computation time from several days to 3 h.

Finally, the docking approach using Ray Casting [88] allows a virtual screening by docking small molecules into protein surface pockets; it can be used to identify known inhibitors from large sets of decoy compounds and new compounds that are active in biochemical assays. Compared with the CPU-based counterpart, the execution on a mid-range price GPU allowed a 27× speed-up.

Table 4 lists the GPU-enabled molecular docking tools described in this section.

**Table 4:** GPU-powered tools for molecular docking, along with the speed-up achieved and the solutions used for code parallelization

Molecular docking				
	Tool name	Speed-up	Parallel solution	Reference
*Hex* spherical polar Fourier protein docking algorithm for rigid molecules	–	45×	CPU-GPU	[80]
Conformation generation and scoring function for rigid and flexible molecules	–	50×	CPU-GPU	[82]
High accuracy flexible molecular docking with differential evolution	MolDock	27.4×	GPU	[83]
Large-scale protein structure alignment	ppsAlign	39×	CPU-GPU	[84]
Protein-DNA docking with Monte Carlo simulation and simulated annealing	–	28×	GPU	[85]
Katchalski-Katzir algorithm with traditional Fast Fourier transform rigid- docking scheme	MEGADOCK	–	GPU	[86]
Docking approach using Ray Casting	–	27×	CPU-GPU	[88]

## Prediction and searching of molecular structures

The computation of secondary structures of RNA or single-stranded DNA molecules is based on the identification of stable, minimum free-energy configurations. Rizk and Lavenier [89] introduced a GPU-accelerated tool based on dynamic programming for the inference of the secondary structure of unfolded RNA [90], adapted from the UNAFold package [91], achieving a 17× speed-up with respect to sequential execution. Similarly, Lei *et al.* [92] proposed a tool based on the Zucker algorithm, which exploits a heterogeneous computing model able to distribute the calculations over multiple threads on both CPU and GPU. The source for these implementations was highly optimized: the performances of CPU code were improved by leveraging both SSE and multi-threading (using the OpenMP library), while the GPU code was optimized by strongly improving the use of the memory hierarchy. Tested on a machine equipped with a quad core CPU Intel Xeon E5620 2.4 GHz, and a GPU Nvidia Geforce GTX 580, the authors experienced a 15.93× speed-up on relatively small sequences (120 bases). However, in the case of longer sequences (e.g. 221 bases), the speed-up drops down to 6.75×.

For the inference of the tertiary structure of proteins, molecular dynamics could be (in principle) exploited as a basic methodology; however, this strategy is usually unfeasible because of the huge computational costs. MemHPG is a memetic hybrid methodology [93], which combines Particle Swarm Optimization [94] and the crossover mechanism typical of evolutionary algorithms to calculate the three-dimensional structure of a target protein, according to (possibly) incomplete data measured with NMR experiments. Thanks to GPU parallelization, used to distribute the calculations of inter-atomic distances for all candidate solutions, the computational cost of the methodology was strongly reduced [95].

Another issue in structural Computational Biology is related to the identification of proteins in databases, according to their three-dimensional conformation. The similarity between two molecules is generally assessed by means of structural alignment, which is characterized by a high computational complexity. GPU-CASSERT [96] mitigates the problem with GPUs, performing a two-phase alignment of protein structures with an average 180× speed-up with respect to its CPU-bound and single-core implementation.

Another methodology for protein searching, working at the level of secondary structures, was proposed by Stivala *et al.* [97]. In this work, the authors performed multiple parallel instances of simulated annealing on the GPU, strongly reducing the computational effort and obtaining a fast methodology that is comparable in accuracy with the state-of-the-art methods.

Table 5 lists the tools presented in this section, along with the speed-up obtained.

**Table 5:** GPU-powered tools to predict molecular structures, along with the speed-up achieved and the solutions used for code parallelization

Prediction and searching of molecular structures				
	Tool name	Speed-up	Parallel solution	Reference
RNA secondary structure with dynamic programming	–	17×	GPU	[89]
RNA secondary structure with Zucker algorithm	–	6.75–15.93×	CPU-GPU	[92]
Molecular distance geometry problem with a memetic algorithm	memHPG	–	CPU-GPU	[93]
Protein alignment	GPU-CASSERT	180×	GPU	[96]
Protein alignment based on Simulated Annealing	–	–	GPU	[97]

## Simulation of spatio-temporal dynamics

The simulation of mathematical models describing complex biological systems allows to determine the quantitative variation of the molecular species in time and in space. Simulations can be performed by means of deterministic, stochastic or hybrid algorithms [98], which should be chosen according to the scale of the modeled system, the nature of its components and the possible role played by biological noise. In this section, we review GPU-powered tools for the simulation of spatio-temporal dynamics and related applications in Systems Biology (see also Table 6).

**Table 6:** GPU-powered tools for dynamic simulation, along with the speed-up achieved and the solutions used for code parallelization

Simulation of the spatio-temporal dynamics and applications in Systems Biology				
	Tool name	Speed-up	Parallel solution	Reference
Coarse-grain deterministic simulation with Euler method	–	63×	GPU	[99]
Coarse-grain deterministic simulation with LSODA	cupSODA	86×	GPU	[100]
Coarse-grain deterministic and stochastic simulation with LSODA and SSA	cuda-sim	47×	GPU	[101]
Coarse-grain stochastic simulation with SSA (with CUDA implementation of Mersenne-Twister RNG)	–	50×	GPU	[102]
Coarse- and fine-grain stochastic simulation with SSA	–	130×	GPU	[103]
Coarse-grain stochastic simulation with SSA	–	–	GPU	[104]
Fine-grain stochastic simulation of large scale models with SSA	GPU-ODM	–	GPU	[105]
Fine-grain stochastic simulation with τ-leaping	–	60×	GPU	[106]
Coarse-grain stochastic simulation with τ-leaping	cuTauLeaping	1000×	GPU	[107]
RD simulation with SSA	–	–	GPU	[108]
Spatial τ-leaping simulation for crowded compartments	STAUCC	24×	GPU	[109]
Particle-based methods for crowded compartments	–	200×	GPU	[110]
Particle-based methods for crowded compartments	–	135×	GPU	[111]
ABM for cellular level dynamics	FLAME	–	GPU	[112]
ABM for cellular level dynamics	–	100×	GPU	[113]
Coarse-grain deterministic simulation of blood coagulation cascade	coagSODA	181×	GPU	[114]
Simulation of large-scale models with LSODA	cupSODA*L	–	GPU	[115]
Parameter estimation with multi-swarm PSO	–	24×	GPU	[116]
Reverse engineering with Cartesian Genetic Programming	cuRE	–	GPU	[95]
Parameter estimation and model selection with approximate Bayesian computation	ABC-SysBio	–	GPU	[117]

### Deterministic simulation

When the concentrations of molecular species are high and the effect of noise can be neglected, Ordinary Differential Equations (ODEs) represent the typical modeling approach for biological systems. Given a model parameterization (i.e. the initial state of the system and the set of kinetic parameters), the dynamics of the system can be obtained by solving the ODEs using some numerical integrator [118].

Ackermann *et al.* [99] developed a GPU-accelerated simulator to execute massively parallel simulations of biological molecular networks. This methodology automatically converts a model, described using the SBML language [119], into a specific CUDA implementation of the Euler numerical integrator. The CPU code used to test this simulator was completely identical to the CUDA code, without any GPU-specific statements; specifically, no multi-threading or SIMD instructions were exploited. The evaluation of this implementation on a Nvidia GeForce 9800 GX2 showed a speed-up between 28× and 63×, compared with the execution on a CPU Xeon 2.66 GHz. In a similar vein, a CUDA implementation of the LSODA algorithm, named cuda-sim, was presented by Zhou *et al.* [101]. LSODA is a numeric integration algorithm that allows higher-quality simulations with respect to Euler’s method, and accelerates the computation also in the case of stiff systems [120]. The cuda-sim simulator performs the so-called ‘just in time’ (JIT) compilation (that is, the creation, compilation and linking at ‘runtime’ of new source code) by converting a SBML model into CUDA code. With respect to the CPU implementation of LSODA contained in the numpy library of Python, cuda-sim achieved a 47× speed-up.

Nobile *et al.* [100] presented another parallel simulator relying on the LSODA algorithm, named cupSODA, to speed up the simultaneous execution of a large number of deterministic simulations. Given a reaction-based mechanistic model and assuming the mass-action kinetics, cupSODA automatically determines the corresponding system of ODEs and the related Jacobian matrix. Differently from cuda-sim, cupSODA saves execution time by avoiding JIT compilation and by relying on a GPU-side parser. cupSODA achieved an acceleration up to 86× with respect to COPASI [121], used as reference CPU-based LSODA simulator. This relevant acceleration was obtained, thanks to a meticulous optimization of the data structures and an intensive usage of the whole memory hierarchy on GPUs (e.g. the ODEs and the Jacobian matrix are stored in the constant memory, while the state of the system is stored in the shared memory). As an extension of cupSODA, coagSODA [114] was then designed to accelerate parallel simulations of a model of the blood coagulation cascade [122], which requires the integration of ODEs based on Hill kinetics, while cupSODA*L [115] was specifically designed to simulate large-scale models (characterized by thousands reactions), which have huge memory requirements owing to LSODA’s working data structures.

### Stochastic simulation

When the effect of biological noise cannot be neglected, randomness can be described either by means of Stochastic Differential Equations [123] or using explicit mechanistic models, whereby the biochemical reactions that describe the physical interactions between the species occurring in the system are specified [124]. In this case, the simulation is performed by means of Monte Carlo procedures, like the stochastic simulation algorithm (SSA) [124].

A problematic issue in the execution of stochastic simulations is the availability of GPU-side high-quality random number generators (RNGs). Although the last versions of CUDA offer the CURAND library (see Supplementary File 2), early GPU implementations required the development of custom kernels for RNGs. This problem was faced for the CUDA version of SSA developed by Li and Petzold [102], who implemented the Mersenne Twister RNG [125], achieving a 50× speed-up with respect to a common single-threaded CPU implementation of SSA. Sumiyoshi *et al.* [103] extended this methodology by performing both coarse-grain and fine-grain parallelization: the former allows multiple simultaneous stochastic simulations of a model, while the latter is achieved by distributing over multiple threads the calculations related to the model reactions. The execution of SSA was optimized by storing both the system state and the values of propensity functions into the shared memory, and by exploiting asynchronous data transfer from the GPU to the CPU to reduce the transfer time. This version of SSA achieved a 130× speed-up with respect to the sequential simulation on the host computer.

Klingbeil *et al.* [104] investigated two different parallelization strategies for coarse-grain simulation with SSA: ‘fat’ and ‘thin’ threads, respectively. The former approach aims at maximizing the usage of shared memory and registers to reduce the data access time; the latter approach exploits lightweight kernels to maximize the number of parallel threads. By testing the two approaches on various models of increasing complexity, the authors showed that ‘fat’ threads are more convenient only in the case of small-scale models owing to the scarcity of the shared memory. Komarov and D’Souza [105] designed GPU-ODM, a fine-grain simulator of large-scale models based on SSA, which makes a clever use of CUDA warp voting functionalities (see Supplementary File 2) and special data structures to efficiently distribute the calculations over multiple threads. Thanks to these optimizations, GPU-ODM outperformed the most advanced (even multi-threaded) CPU-based implementations of SSA.

The τ-leaping algorithm allows a faster generation of the dynamics of stochastic models with respect to SSA, by properly calculating longer simulation steps [126, 127]. Komarov *et al.* [106] proposed a GPU-powered fine-grain τ-leaping implementation, which was shown to be efficient in the case of extremely large (synthetic) biochemical networks (i.e. characterized by >10^5^ reactions). Nobile *et al.* [107] then proposed cuTauLeaping, a GPU-powered coarse-grain implementation of the optimized version of τ-leaping proposed by Cao *et al.* [127]. Thanks to the optimization of data structures in low-latency memories, to the use of warp voting and to the splitting of the algorithm into multiple phases corresponding to lightweight CUDA kernels, cuTauLeaping was up to three orders of magnitude faster on a GeForce GTX 590 GPU than the CPU-based implementation of τ-leaping contained in COPASI, executed on a CPU Intel Core i7-2600 3.4 GHz.

### Spatial simulation

When the spatial localization or the diffusion of chemical species has a relevant role on the emergent dynamics, biological systems should be modeled by means of Partial Differential Equations (PDEs), thus defining Reaction-Diffusion (RD) models [128]. Several GPU-powered tools for the numerical integration of PDEs have been proposed [129–131].

In the case of stochastic RD models, the simulation is generally performed by partitioning the reaction volume into a set of small sub-volumes, in which the molecular species are assumed to be well-stirred. This allows to exploit extended versions of stochastic simulation algorithms like SSA or τ-leaping, explicitly modified to consider the diffusion of species from one sub-volume toward its neighbors. Vigelius *et al.* [108] presented a GPU-powered simulator of RD models based on SSA. Pasquale *et al.* [109] proposed STAUCC (Spatial Tau-leaping in Crowded Compartments), a GPU-powered simulator of RD models based on the Sτ-DPP algorithm [132], a τ-leaping variant that keeps into account the size of the macromolecules. According to published results [109], STAUCC achieves up to 24× speed-up with respect to the sequential execution.

Smoldyn proposes an alternative approach to stochastic RD models, where molecules are modeled as individual particles [133]. Although species move stochastically, reactions are fired deterministically; in the case of second-order reactions, two particles react when they are close enough to collide. Two GPU-accelerated versions of Smoldyn were proposed by Gladkov *et al.* [110] and by Dematté [111]. Although the former offers a greater acceleration (i.e. 200×), the latter shows another peculiarity of GPUs: the graphics interoperability, that is, the possibility of plotting the positions of particles in real time, by accessing the system state that resides on GPU’s global memory.

By changing the modeling paradigm, agent-based models (ABMs) explicitly represent the individual actors of a complex system (e.g. cells), tracking their information throughout a simulation. FLAME [112] is a general-purpose simulator of ABMs, which exploits GPU acceleration to strongly reduce the running time. It is worth noting that an alternative parallelization of ABMs by means of grid computing would not scale well: the running time could not be reduced below a fixed threshold—even by increasing the number of processors—because of memory bandwidth restrictions, which do not occur in the case of GPU acceleration [112]. A tailored GPU-powered simulator of ABMs was also developed by D’Souza *et al.* [113], to accelerate the investigation of tuberculosis.

A final issue worth to be mentioned is the multi-scale simulation of biological systems, ranging from intracellular gene regulation up to cell shaping, adhesion and movement. For instance, Christley *et al.* [134] proposed a method for the investigation of epidermal growth model, which fully leveraged GPU’s horsepower by breaking the simulation into smaller kernels and by adopting GPU-tailored data structures.

### Applications in Systems Biology

The computational methods used in Systems Biology to perform thorough analyses of biological systems—such as sensitivity analysis, parameter estimation, parameter sweep analysis [135, 136]—generally rely on the execution of a large number of simulations to explore the high-dimensional search space of possible model parameterizations. The aforementioned GPU-accelerated simulators can be exploited to reduce the huge computational costs of these analyses.

For instance, cuTauLeaping [107] was applied to carry out a bi-dimensional parameter sweep analysis to analyze the insurgence of oscillatory regimes in a glucose-dependent signal transduction pathway in yeast. Thanks to the GPU acceleration, 2^16^ stochastic simulations—corresponding to 2^16^ different parameterizations of the model—were executed in parallel in just 2 h. coagSODA [114] was exploited to execute one-dimensional and bi-dimensional parameter sweep analyses of a large mechanistic model of the blood coagulation cascade, to determine any alteration (prolongation or reduction) of the clotting time in response to perturbed values of reaction constants and of the initial concentration of some pivotal species. The comparison of the running time required to execute a parameter sweep analysis with 10^5^ different parameterizations showed a 181× speed-up on Nvidia Tesla K20c GPU with respect to an Intel Core i5 CPU.

Nobile *et al.* [116] proposed a parameter estimation methodology based on a multi-swarm version of Particle Swarm Optimization (PSO) [94], which exploits a CUDA-powered version of SSA. This method, tailored for the estimation of kinetic constants in stochastic reaction-based models, achieved a 24× speed-up with respect to an equivalent CPU implementation. The tool cuRE [95] integrates this parameter estimation methodology with Cartesian Genetic Programming [137], to perform the reverse engineering of biochemical interaction networks. Liepe *et al.* [117] proposed ABC-SysBio, a Python-based and GPU-powered framework based on approximate Bayesian computation, able to perform both parameter estimation and model selection. ABC-SysBio also represents the foundation for SYSBIONS [138], a tool for the calculation of a model’s evidence and the generation of samples from the posterior parameter distribution.

## Discussion

In this article we reviewed the recent state-of-the-art of GPU-powered tools available for applications in Bioinformatics, Computational Biology and Systems Biology. We highlight here that, although the speed-up values reported in literature confirm that GPUs represent a powerful means to strongly reduce the running times, many of the measured acceleration could be controversial, as there might be room for additional optimization of the code executed on the CPU. Indeed, according to the descriptions provided in the aforementioned papers, many performance tests were performed using CPU code that leverage neither multi-threading nor vectorial instructions (e.g. those offered by SSE [9] or AVX instruction sets [139]). However, some of the reported speed-up values are so relevant—e.g. the 180× acceleration provided by GPU-CASSERT [96], or the 50× acceleration provided by the molecular docking tool developed by Korb *et al.* [82]—that even an optimized CPU code could hardly outperform the CUDA code.

In addition, it is worth noting that many of the most performing tools required a tailored implementation to fully leverage the GPU architecture and its theoretical peak performance. For instance, the fine-/coarse-grain implementation of SSA presented by Sumiyoshi *et al.* [103] relies on the skillful usage of shared memory and asynchronous data transfers; the protein alignment tool GPU-CASSERT [96] relies on a highly optimized use of global memory and multiple streams of execution, overlapped with data transfers; the stochastic simulator cuTauLeaping [107] relies on GPU-optimized data structures, on the fragmentation of the execution into multiple ‘thin’ kernels, and on the crafty usage of both constant and shared memories. These works provide some examples of advanced strategies used in GPGPU computing, which make CUDA implementations far more complicated than classic CPU-bound implementations. In general, the most efficient GPU-powered implementations share the following characteristics: they leverage the high-performance memories, and try to reduce the accesses to the global memory by exploiting GPU-optimized data structures. These features seem to represent the key to successful CUDA implementations, along with optimized memory layouts [140] and a smart partitioning of tasks over several threads with limited branch divergence. Stated otherwise, we warn that a naïve porting of an existing software to CUDA is generally doomed to failure.

As previously mentioned, CUDA is by far the most used library for GPGPU computing; anyway, alternative solutions exist. OpenCL, for instance, is an open standard suitable for parallel programming of heterogeneous systems [141]; it includes an abstract model for architecture and memory hierarchy of OpenCL-compliant computing devices, a C-like programming language for the device-side code and C API (Application Programming Interface) for the host-side. The execution and memory hierarchy models of OpenCL are similar to CUDA, as OpenCL exploits a dedicated compiler to appropriately compile kernels according to the available devices. Differently from CUDA, the kernel compilation phase of OpenCL is performed at runtime. However, CUDA 7.0 introduced this possibility with the NVRTC library [142]. The difficulty in writing code with OpenCL led to the definition of tools as Swan [143], to facilitate the porting of existing CUDA code to OpenCL and minimizing the effort of code rewriting. The performances of CUDA code and OpenCL code converted with Swan have been compared [143], showing a 50% increment of the execution time of the OpenCL version: the CUDA compiler appeared to be more efficient in reducing registers usage, which affects the number of concurrently executed threads. In addition, the kernel launch cost of OpenCL is around nine times larger than CUDA, affecting the running time especially in the case of kernels with ‘short’ execution time.

On the contrary, an interesting feature of Swan [143] is that CUDA code ported to OpenCL was successfully executed both on Nvidia and AMD devices without any changes to the source code, making this tool an appealing alternative to full re-implementation. Hence, although CUDA-optimized code is still more efficient [140]—see e.g. the case of MaxSSmap [31], where the source code compiled with the last versions of the CUDA library largely outperforms OpenCL—the OpenCL library represents a viable alternative to CUDA, as it is hardware independent, and it can reduce the costs of porting and maintaining multi-platform support of applications.

**Figure 1:**
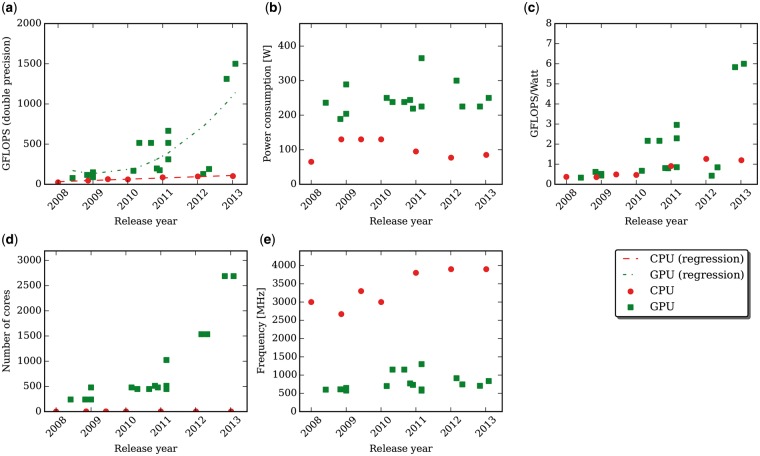
With the advances in the manufacturing processes, the architectural features of both CPUs (red dots) and GPUs (green squares) continuously improve. This figure shows the trends for both architectures by comparing the following characteristics: (**A**) the performances in terms of GFLOPS when performing double precision floating point operations; (**B**) the power consumption; (**C**) the GPWR; (**D**) the number of cores per unit; (**E**) the core working frequencies. The GPUs considered in this figure are reported in Supplementary File 3, while the CPUs are the Intel Core i7 processors released in the same years (namely, from the Westmere up to the Haswell microarchitectures). A colour version of this figure is available at BIB online: https://academic.oup.com/bib.

Although the speed-up achieved with optimized CUDA code is already relevant, it is worth noting that the constant improvement in the fabrication process of GPU-enabled video cards is expected to further increase the efficiency gap with respect to CPUs. The speed-up of GPU-powered software is generally higher when running the code on more recent video cards, thanks to the larger number of cores and the increment of the available high-performance resources (e.g. registers, shared memory, cache), which remove the main limitations to a full occupancy of GPUs in many existing implementations. Figure 1 summarizes some general trends of CPUs (red dots) and of the GPUs (green squares) that were cited in this review and that are listed in Supplementary File 3.

Figure 1a compares the theoretical GFLOPS performance assuming double precision floating point calculations: even though both architectures are constantly improving, GPUs’ performances enhance at a faster rate (as shown by the regression lines), with the most recent architectures being almost two orders of magnitude more efficient than CPUs. Higher performances are directly reflected in higher energy requirements: Figure 1b compares the energy consumption of the two architectures. The GFLOPS-per-Watt ratio (GPWR, Figure 1c), however, represents a better measure of the efficiency of the devices than the mere power consumption: GPUs generally allow better theoretical performances with respect to CPUs, despite the higher energetic requirements. The higher GPWR of GPUs is the rationale behind the development of GPU-based supercomputers, which represents a ‘green’ alternative to conventional HPC infrastructures. Figure 1d shows that, nowadays, GPUs largely outnumber CPUs, considering the number of cores, thanks to their exponential increase on the most recent video cards. This characteristic is counterbalanced by the far lower working frequency of video cards (Figure 1e), although even CPUs frequency did not substantially improve in the last years. These data explain why GPU-powered software, which leverage the thousands of cores contained in a GPU, are expected to experience a relevant increment in the achievable speed-up if they are executed on newer architectures.

A potential drawback of GPUs is the availability of memory. As a matter of fact, many applications—in particular those processing genome-wide data—require a huge amount of memory, more than the few gigabytes contained on high-end GPUs at the time of writing. From the point of view of memory, CPUs still largely outperform GPUs. However, CUDA allows kernels to directly access CPU’s RAM by means of the so-called ‘pinned memory’ [144]. This type of memory is page-locked and can be directly read and written from the GPU, using Direct Memory Access through the PCI-express bus, without any involvement of the CPU. The drawback of this solution is represented by the bandwidth of PCI-express accesses, which provides a reduced rate with respect to device-to-device memory transfers [145]. cupSODA*L [115] is one example of computational tool following this strategy, where the pinned memory was leveraged to perform coarse-grain simulation of large-scale biochemical models achieving only a limited speed-up.

Taking all of these issues into consideration, it can be anticipated that the increasing availability of GPU-powered tools in various research areas of life sciences—as well as the creation of massive GPU-based infrastructures, providing scientists with hexa-scale performances—will finally enable the execution of fastest and thorough simulations and analyses of complex molecular structures, or pave the way to ambitious goals like genome-wide analyses and dynamical simulations of detailed mechanistic models of whole cells and organisms.

Key Points

- Computational methods and software tools developed in Bioinformatics, Computational Biology and Systems Biology can be computationally demanding when executed on Central Processing Units (CPUs), therefore limiting their applicability in many circumstances.
- General-purpose Graphics Processing Units (GPUs) are nowadays gaining an increasing attention by the scientific community, as they can considerably reduce the running time required by standard CPU-based software.
- The aim of this review is to provide an overview of recent GPU-powered tools developed in Bioinformatics, Computational Biology and Systems Biology, emphasizing their advantages (i.e. computational speed-up) as well as drawbacks (e.g. the necessity of algorithm redesign and tailored implementation to fully leverage the GPU architecture and its peak performance).
- In particular, we present recent GPU-accelerated methodologies developed for sequence alignment, molecular dynamics, molecular docking, prediction and searching of molecular structures, simulation of the spatio-temporal dynamics of cellular processes and related applications in Systems Biology.
- The main concepts related to GPUs, a collection of other applications in Bioinformatics and Computational Biology (spectral analysis, genome-wide analysis, Bayesian inference, movement tracking, quantum chemistry) and additional technical details about Nvidia GPUs are provided in the supplementary files.

## Supplementary data

Supplementary data are available online at http://bib.oxfordjournals.org/.

## Supplementary Material

Supplementary Data

Supplementary Data

Supplementary Data

## Notes

**Marco S. Nobile** (M.Sc. and Ph.D. in Computer Science) is Research Fellow at the University of Milano-Bicocca, Italy, since January 2015. His research interests concern high-performance computing, evolutionary computation, Systems Biology.

## Notes

**Paolo Cazzaniga** (M.Sc. and Ph.D. in Computer Science) is Assistant Professor at the University of Bergamo, Italy, since February 2011. His research interests concern computational intelligence, Systems Biology, high-performance computing.

## Notes

**Andrea Tangherloni** (M.Sc. in Computer Science) is PhD student in Computer Science at the University of Milano-Bicocca, Italy, since December 2015. His research interests concern high-performance computing and swarm intelligence.

## Notes

**Daniela Besozzi** (M.Sc. in Mathematics, Ph.D. in Computer Science) is Associate Professor at the University of Milano-Bicocca, Italy, since October 2015. Her research interests concern mathematical modeling, Systems Biology, Computational Biology.

## References

[1] Rapaport DC. The Art of Molecular Dynamics Simulation. Cambridge: Cambridge University Press, 2004.

[2] Haile JM. Molecular Dynamics Simulation: Elementary Methods. New York: Wiley, 1997.

[3] He M, Petoukhov S. Mathematics of Bioinformatics: Theory, Methods and Applications. Hoboken, NJ: John Wiley & Sons, 2011.

[4] Alberghina L, Westerhoff HV. Systems Biology: Definitions and Perspectives, Vol. 13 of Topics in Current Genetics. Berlin, Germany: Springer-Verlag, 2005.

[5] Karr JR, Sanghvi JC, Macklin DN, et al A whole-cell computational model predicts phenotype from genotype. Cell 2012;150(2):389–401. 22817898 10.1016/j.cell.2012.05.044 PMC3413483

[6] Schulz R, Lindner B, Petridis L, et al Scaling of multimillion-atom biological molecular dynamics simulation on a petascale supercomputer. J Chem Theory Comput 2009;5(10):2798–808. 26631792 10.1021/ct900292r

[7] Sauro HM, Harel D, Kwiatkowska M, et al Challenges for modeling and simulation methods in systems biology In: Perrone L, Wieland F, Liu J, et al (eds). Proceedings of the 38th Conference on Winter Simulation. New York: IEEE, 2006, 1720–30.

[8] Pop M, Salzberg SL. Bioinformatics challenges of new sequencing technology. Trends Genet 2008;24(3):142–9. 18262676 10.1016/j.tig.2007.12.007 PMC2680276

[9] Intel® SSE4 Programming Reference. Reference Number: D91561-003. Intel Corporation, Denver, CO, USA, 2007. Available at: https://software.intel.com/sites/default/files/m/8/b/8/D9156103.pdf.

[10] Iosup A, Epema D. Grid computing workloads. Internet Comput IEEE 2011;15(2):19–26.

[11] Foster I, Kesselman C. The Grid 2: Blueprint for a New Computing Infrastructure. Los Alamitos, CA: Elsevier, 2003.

[12] Armbrust M, Fox A, Griffith R, et al A view of cloud computing. Commun ACM 2010;53(4):50–8.

[13] Sarkar S, Majumder T, Kalyanaraman A, et al Hardware accelerators for biocomputing: a survey In: Proceedings of 2010 IEEE International Symposium on Circuits and Systems (ISCAS). Reston, VA: IEEE, 2010, 3789–92.

[14] Joubert W, Archibald R, Berrill M, et al Accelerated application development: The ORNL Titan experience. Comput Electr Eng 2015;46:123–38.

[15] Bland AS, Wells JC, Messer OE, et al Titan: early experience with the Cray XK6 at Oak Ridge National Laboratory In: Proceedings of Cray User Group Conference (CUG 2012). Stuttgart, Germany: Cray User Group, 2012.

[16] Amdahl GM. Validity of the single processor approach to achieving large scale computing capabilities In: AFIPS '67 (Spring) Proceedings of the April 18–20, 1967, Spring Joint Computer Conference. New York: ACM, 1967, 483–5.

[17] Farber RM. Topical perspective on massive threading and parallelism. J Mol Graphics Model 2011;30:82–9. 10.1016/j.jmgm.2011.06.007 21764615

[18] Che S, Li J, Sheaffer JW, et al Accelerating compute-intensive applications with GPUs and FPGAs In: Symposium on Application Specific Processors, 2008. SASP 2008. Washington, DC: IEEE, 2008, 101–7.

[19] Dematté L, Prandi D. GPU computing for systems biology. Brief Bioinform 2010;11(3):323–33. 20211843 10.1093/bib/bbq006

[20] Harvey MJ, De Fabritiis G. A survey of computational molecular science using graphics processing units. WIREs Comput Mol Sci 2012;2(5):734–42.

[21] Payne JL, Sinnott-Armstrong NA, Moore JH. Exploiting graphics processing units for computational biology and bioinformatics. Interdiscipl Sci Comput Life 2010;2(3):213–20. 10.1007/s12539-010-0002-4 PMC2910913 20658333

[22] Klus P, Lam S, Lyberg D, et al BarraCUDA—a fast short read sequence aligner using graphics processing units. BMC Res Notes 2012;5:27. 22244497 10.1186/1756-0500-5-27 PMC3278344

[23] Liu Y, Schmidt B, Maskell DL. CUSHAW: a CUDA compatible short read aligner to large genomes based on the Burrows–Wheeler transform. Bioinformatics 2012;28(14):1830–7. 22576173 10.1093/bioinformatics/bts276

[24] Torres JS, Espert IB, Dominguez AT, et al Using GPUs for the exact alignment of short-read genetic sequences by means of the Burrows-Wheeler transform. IEEE/ACM Trans Comput Biol Bioinform 2012;9(4):1245–56. 22450827 10.1109/TCBB.2012.49

[25] Liu CM, Wong T, Wu E, et al SOAP3: ultra-fast GPU-based parallel alignment tool for short reads. Bioinformatics 2012;28(6):878–9. 22285832 10.1093/bioinformatics/bts061

[26] Blom J, Jakobi T, Doppmeier D, et al Exact and complete short-read alignment to microbial genomes using Graphics Processing Unit programming. Bioinformatics 2011;27(10):1351–8. 21450712 10.1093/bioinformatics/btr151

[27] Luo R, Wong T, Zhu J, et al SOAP3-dp: fast, accurate and sensitive GPU-based short read aligner. PLoS One 2013;8(5):e65632. 23741504 10.1371/journal.pone.0065632 PMC3669295

[28] Manconi A, Orro A, Manca E, et al A tool for mapping single nucleotide polymorphisms using Graphics Processing Units. BMC Bioinformatics 2014;15(Suppl 1):S10. 10.1186/1471-2105-15-S1-S10 PMC4015528 24564714

[29] Manconi A, Manca E, Moscatelli M, et al G-CNV: a GPU-based tool for preparing data to detect CNVs with read-depth methods. Front Bioeng Biotechnol 2015;3:28. 25806367 10.3389/fbioe.2015.00028 PMC4354384

[30] Langmead B, Salzberg SL. Fast gapped-read alignment with Bowtie 2. Nat Methods 2012;9(4):357–9. 22388286 10.1038/nmeth.1923 PMC3322381

[31] Turki T, Roshan U. MaxSSmap: a GPU program for mapping divergent short reads to genomes with the maximum scoring subsequence. BMC Genomics 2014;15:969. 25398475 10.1186/1471-2164-15-969 PMC4289234

[32] Mahmood SF, Rangwala H. GPU-Euler: sequence assembly using GPGPU In: IEEE 13th International Conference on High Performance Computing and Communications (HPCC), 2011. Los Alamitos, CA: IEEE, 2011, 153–60.

[33] Li D, Liu CM, Luo R, et al MEGAHIT: an ultra-fast single-node solution for large and complex metagenomics assembly via succinct de Bruijn graph. Bioinformatics 2015;31(10):1674–6. 25609793 10.1093/bioinformatics/btv033

[34] Ling C, Benkrid K. Design and implementation of a CUDA-compatible GPU-based core for gapped BLAST algorithm. Procedia Comput Sci 2010;1(1):495–504.

[35] Liu W, Schmidt B, Muller-Wittig W. CUDA-BLASTP: accelerating BLASTP on CUDA-enabled graphics hardware. IEEE/ACM Trans Comput Biol Bioinform 2011;8(6):1678–84. 21339531 10.1109/TCBB.2011.33

[36] Zhao K, Chu X. G-BLASTN: Accelerating nucleotide alignment by graphics processors. Bioinformatics 2014;30(10):1384–91. 24463183 10.1093/bioinformatics/btu047

[37] Korpar M, Šikić M. SW# – GPU-enabled exact alignments on genome scale. Bioinformatics 2013;29(19):2494–5. 23864730 10.1093/bioinformatics/btt410 PMC3777108

[38] Trapnell C, Schatz MC. Optimizing data intensive GPGPU computations for DNA sequence alignment. Parallel Comput 2009;35(8):429–40. 20161021 10.1016/j.parco.2009.05.002 PMC2749273

[39] Papadopoulos A, Kirmitzoglou I, Promponas VJ, et al GPU technology as a platform for accelerating local complexity analysis of protein sequences In: Engineering in Medicine and Biology Society (EMBC), 2013 35th Annual International Conference of the IEEE. IEEE, 2013, 2684–7. 10.1109/EMBC.2013.6610093 24110280

[40] Jiang H, Ganesan N. CUDAMPF: a multi-tiered parallel framework for accelerating protein sequence search in HMMER on CUDA-enabled GPU. BMC Bioinformatics 2016;17:106. 26920848 10.1186/s12859-016-0946-4 PMC4769571

[41] Pham HP, Nguyen HD, Nguyen TT. Aligning multi sequences on GPUs In Vinh PC, Hung NM, Tung NT, et al (eds). Context-Aware Systems and Applications, Vol. 109 of Lecture Notes of the Institute for Computer Sciences, Social Informatics and Telecommunications Engineering. Heidelberg, Berlin: Springer, 2013, 300–9.

[42] Lin CY, Lin YS. Efficient parallel algorithm for multiple sequence alignments with regular expression constraints on graphics processing units. Int J Comput Sci Eng 2014;9(1):11–20.

[43] Burrows M, Wheeler DJ. A block-sorting loss-less data compression algorithm. Technical report. Palo Alto, CA: Digital Equipment Corporation, 1994.

[44] Ferragina P, Manzini G. Indexing compressed text. J ACM 2005;52(4):552–81.

[45] NVBIO webpage. http://nvlabs.github.io/nvbio/.

[46] Altschul SF, Gish W, Miller W, et al Basic local alignment search tool. J Mol Biol 1990;215(3):403–10. 2231712 10.1016/S0022-2836(05)80360-2

[47] Altschul SF, Madden TL, Schäffer AA, et al Gapped BLAST and PSI-BLAST: a new generation of protein database search programs. Nucleic Acids Res 1997;25(17):3389–402. 9254694 10.1093/nar/25.17.3389 PMC146917

[48] Smith TF, Waterman MS. Identification of common molecular subsequences. J Mol Biol 1981;147(1):195–7. 7265238 10.1016/0022-2836(81)90087-5

[49] Liu Y, Wirawan A, Schmidt B. CUDASW ++ 3.0: accelerating Smith-Waterman protein database search by coupling CPU and GPU SIMD instructions. BMC Bioinformatics 2013;14:117. 23557111 10.1186/1471-2105-14-117 PMC3637623

[50] Manavski SA, Valle G. CUDA compatible GPU cards as efficient hardware accelerators for Smith-Waterman sequence alignment. BMC Bioinformatics 2008;9(Suppl 2):S10. 10.1186/1471-2105-9-S2-S10 PMC2323659 18387198

[51] Liu Y, Maskell DL, Schmidt B. CUDASW ++: optimizing Smith-Waterman sequence database searches for CUDA-enabled graphics processing units. BMC Res Notes 2009;2:73. 19416548 10.1186/1756-0500-2-73 PMC2694204

[52] Liu Y, Schmidt B, Maskell DL. CUDASW ++ 2.0: enhanced Smith-Waterman protein database search on CUDA-enabled GPUs based on SIMT and virtualized SIMD abstractions. BMC Res Notes 2010;3:93. 20370891 10.1186/1756-0500-3-93 PMC2907862

[53] Rognes T. Faster Smith-Waterman database searches with inter-sequence SIMD parallelisation. BMC Bioinformatics 2011;12(221) 10.1186/1471-2105-12-221 PMC3120707 21631914

[54] Camacho C, Coulouris G, Avagyan V, et al BLAST+: architecture and applications. BMC Bioinformatics 2009;10:421. 20003500 10.1186/1471-2105-10-421 PMC2803857

[55] Delcher AL, Phillippy A, Carlton J, et al Fast algorithms for large-scale genome alignment and comparison. Nucleic Acids Res 2002;30(11):2478–83. 12034836 10.1093/nar/30.11.2478 PMC117189

[56] Promponas VJ, Enright AJ, Tsoka S, et al CAST: an iterative algorithm for the complexity analysis of sequence tracts. Bioinformatics 2000;16(10):915–22. 11120681 10.1093/bioinformatics/16.10.915

[57] Krogh A, Brown M, Mian IS, et al Hidden Markov models in computational biology: Applications to protein modeling. J Mol Biol 1994;235(5):1501–31. 8107089 10.1006/jmbi.1994.1104

[58] Ganesan N, Chamberlain RD, Buhler J, et al Accelerating HMMER on GPUs by implementing hybrid data and task parallelism In: Proceedings of the First ACM International Conference on Bioinformatics and Computational Biology. New York: ACM, 2010, 418–21.

[59] Chenna R, Sugawara H, Koike T, et al Multiple sequence alignment with the Clustal series of programs. Nucleic Acids Res 2003;31(13):3497–500. 12824352 10.1093/nar/gkg500 PMC168907

[60] Smock RG, Gierasch LM. Sending signals dynamically. Science 2009;324(5924):198–203. 19359576 10.1126/science.1169377 PMC2921701

[61] McCammon JA, Gelin BR, Karplus M. Dynamics of folded proteins. Nature 1977;267(5612):585–90. 301613 10.1038/267585a0

[62] Klepeis JL, Lindorff-Larsen K, Dror RO, et al Long-timescale molecular dynamics simulations of protein structure and function. Curr Opin Struct Biol 2009;19(2):120–7. 19361980 10.1016/j.sbi.2009.03.004

[63] Freddolino PL, Arkhipov AS, Larson SB, et al Molecular dynamics simulations of the complete satellite tobacco mosaic virus. Structure 2006;14(3):437–49. 16531228 10.1016/j.str.2005.11.014

[64] Susukita R, Ebisuzaki T, Elmegreen BG, et al Hardware accelerator for molecular dynamics: MDGRAPE-2. Comput Phys Commun 2003;155(2):115–31.

[65] Shaw DE, Deneroff MM, Dror RO, et al Anton, a special-purpose machine for molecular dynamics simulation. Commun ACM 2008;51(7):91–7.

[66] Liu W, Schmidt B, Voss G, et al Accelerating molecular dynamics simulations using Graphics Processing Units with CUDA. Comput Phys Commun 2008;179(9):634–41.

[67] Salomon-Ferrer R, Götz AW, Poole D, et al Routine microsecond molecular dynamics simulations with AMBER on GPUs. 2. Explicit solvent particle mesh Ewald. J Chem Theory Comput 2013;9(9):3878–88. 26592383 10.1021/ct400314y

[68] Mashimo T, Fukunishi Y, Kamiya N, et al Molecular dynamics simulations accelerated by GPU for biological macromolecules with a non-Ewald scheme for electrostatic interactions. J Chem Theory Comput 2013;9(12):5599–609. 26592294 10.1021/ct400342e

[69] Eastman P, Friedrichs MS, Chodera JD, et al OpenMM 4: a reusable, extensible, hardware independent library for high performance molecular simulation. J Chem Theory Comput 2012;9(1):461–9. 23316124 10.1021/ct300857j PMC3539733

[70] Kylasa SB, Aktulga HM, Grama AY. PuReMD-GPU: a reactive molecular dynamics simulation package for GPUs. J Comput Phys 2014;272:343–59.

[71] Ruymgaart AP, Cardenas AE, Elber R. MOIL-opt: Energy-conserving molecular dynamics on a GPU/CPU system. J Chem Theory Comput 2011;7(10):3072–82. 22328867 10.1021/ct200360f PMC3274753

[72] Sweet JC, Nowling RJ, Cickovski T, et al Long timestep molecular dynamics on the graphical processing unit. J Chem Theory Comput 2013;9(8):3267–81. 24436689 10.1021/ct400331r PMC3890418

[73] Rovigatti L, Šulc P, Reguly IZ, et al A comparison between parallelization approaches in molecular dynamics simulations on GPUs. J Comput Chem 2015;36(1):1–8. 25355527 10.1002/jcc.23763

[74] Le Grand S, Götz AW, Walker RC. SPFP: Speed without compromise – A mixed precision model for GPU accelerated molecular dynamics simulations. Comput Phys Commun 2013;184(2):374–80.

[75] Phillips CL, Anderson JA, Glotzer SC. Pseudo-random number generation for Brownian dynamics and dissipative particle dynamics simulations on GPU devices. J Comput Phys 2011;230(19):7191–201.

[76] Tamascelli D, Dambrosio FS, Conte R, et al Graphics processing units accelerated semiclassical initial value representation molecular dynamics. J Chem Phys 2014;140(17):174109. 24811627 10.1063/1.4873137

[77] Loukatou S, Papageorgiou L, Fakourelis P, et al Molecular dynamics simulations through GPU video games technologies. J Mol Biochem 2014;3(2):64–71. 27525251 PMC4980074

[78] Halperin I, Ma B, Wolfson H, et al Principles of docking: An overview of search algorithms and a guide to scoring functions. Proteins 2002;47(4):409–43. 12001221 10.1002/prot.10115

[79] Grosdidier S, Totrov M, Fernández-Recio J. Computer applications for prediction of protein–protein interactions and rational drug design. Adv Appl Bioinform Chem 2009;2:101. 21918619 PMC3169948

[80] Ritchie DW, Venkatraman V. Ultra-fast FFT protein docking on graphics processors. Bioinformatics 2010;26(19):2398–405. 20685958 10.1093/bioinformatics/btq444

[81] Nvidia. cuFFT library user’s guide 7.5, February 2015 URL: http://docs.nvidia.com/cuda/cufft/.

[82] Korb O, Stutzle T, Exner TE. Accelerating molecular docking calculations using graphics processing units. J Chem Inf Model 2011;51(4):865–76. 21434638 10.1021/ci100459b

[83] Simonsen M, Pedersen CNS, Christensen MH, et al GPU-accelerated high-accuracy molecular docking using guided differential evolution: real world applications In: Proceedings of the 13th Annual Conference on Genetic and Evolutionary Computation. ACM, 2011, 1803–10.

[84] Pang B, Zhao N, Becchi M, et al Accelerating large-scale protein structure alignments with graphics processing units. BMC Res Notes 2012;5:116. 22357132 10.1186/1756-0500-5-116 PMC3309952

[85] Wu J, Chen C, Hong B. A GPU-based approach to accelerate computational protein-DNA docking. Comput Sci Eng 2012;14(3):20–9.

[86] Ohue M, Shimoda T, Suzuki S, et al MEGADOCK 4.0: an ultra–high-performance protein–protein docking software for heterogeneous supercomputers. Bioinformatics 2014;30(22):3281–3. 25100686 10.1093/bioinformatics/btu532 PMC4221127

[87] Kirkpatrick S, Gelatt CD, Vecchi MP. Optimization by simulated annealing. Science 1983;220(4598):671–80. 17813860 10.1126/science.220.4598.671

[88] Khar KR, Goldschmidt L, Karanicolas J. Fast docking on graphics processing units via Ray-Casting. PLoS One 2013;8(8):e70661. 23976948 10.1371/journal.pone.0070661 PMC3745428

[89] Rizk G, Lavenier D. GPU accelerated RNA folding algorithm In Allen G, Nabrzyski J, Seidel E, et al (eds). Computational Science–ICCS 2009. 9th International Conference, 2009 Proceedings, Part I, Vol. 5544 of Lecture Notes in Computer Science. Heidelberg, Berlin: Springer, 2009, 1004–13.

[90] Zuker M, Stiegler P. Optimal computer folding of large RNA sequences using thermodynamics and auxiliary information. Nucleic Acids Res 1981;9(1):133–48. 6163133 10.1093/nar/9.1.133 PMC326673

[91] Markham NR, Zuker M. DINAMelt web server for nucleic acid melting prediction. Nucleic Acids Res 2005;33(2):W577–81. 15980540 10.1093/nar/gki591 PMC1160267

[92] Lei G, Dou Y, Wan W, et al CPU-GPU hybrid accelerating the Zuker algorithm for RNA secondary structure prediction applications. BMC Genomics 2012;13(Suppl 1):S14. 10.1186/1471-2164-13-S1-S14 PMC3303730 22369626

[93] Nobile MS, Citrolo AG, Cazzaniga P, et al A memetic hybrid method for the molecular distance geometry problem with incomplete information In: Proceedings of the 2014 IEEE Congress on Evolutionary Computation (CEC2014). Beijing, China: IEEE, 2014, 1014–21.

[94] Kennedy J, Eberhart RC. Particle swarm optimization. In: Proceedings of the IEEE International Conference on Neural Networks, Vol. **4** Perth, WA: IEEE, 1995, 1942–8.

[95] Nobile MS. Evolutionary inference of biological systems accelerated on Graphics Processing Units. PhD Thesis, University of Milano-Bicocca, Italy, 2015.

[96] Mrozek D, Brożek M, Małysiak-Mrozek B. Parallel implementation of 3D protein structure similarity searches using a GPU and the CUDA. J Mol Model 2014;20(2):1–17. 10.1007/s00894-014-2067-1 PMC3936136 24481593

[97] Stivala AD, Stuckey PJ, Wirth AI. Fast and accurate protein substructure searching with simulated annealing and GPUs. BMC Bioinformatics 2010;11:446. 20813068 10.1186/1471-2105-11-446 PMC2944279

[98] Wilkinson D. Stochastic modelling for quantitative description of heterogeneous biological systems. Nat Rev Genet 2009;10(2):122–33. 19139763 10.1038/nrg2509

[99] Ackermann J, Baecher P, Franzel T, et al Massively-parallel simulation of biochemical systems. In: Proceedings of Massively Parallel Computational Biology on GPUs, Jahrestagung der Gesellschaft für Informatik, e.V, Lübeck, Germany: Lecture Notes in Informatics (LNI), 2009, 739–50.

[100] Nobile MS, Besozzi D, Cazzaniga P, et al GPU-accelerated simulations of mass-action kinetics models with cupSODA. J Supercomput 2014;69(1):17–24.

[101] Zhou Y, Liepe J, Sheng X, et al GPU accelerated biochemical network simulation. Bioinformatics 2011;27(6):874–6. 21224286 10.1093/bioinformatics/btr015 PMC3051321

[102] Li H, Petzold LR. Efficient parallelization of the stochastic simulation algorithm for chemically reacting systems on the Graphics Processing Unit. Int J High Perform Comput Appl 2010;24(2):107–16.

[103] Sumiyoshi K, Hirata K, Hiroi N, et al Acceleration of discrete stochastic biochemical simulation using GPGPU. Front Physiol 2015;6:42. 25762936 10.3389/fphys.2015.00042 PMC4327578

[104] Klingbeil G, Erban R, Giles M, et al Fat versus thin threading approach on GPUs: Application to stochastic simulation of chemical reactions. IEEE Trans Parallel Distrib Syst 2012;23(2):280–7.

[105] Komarov I, D’Souza RM. Accelerating the Gillespie exact stochastic simulation algorithm using hybrid parallel execution on graphics processing units. PLoS One 2012;7(11):e46693. 23152751 10.1371/journal.pone.0046693 PMC3494724

[106] Komarov I, D’Souza RM, Tapia J. Accelerating the Gillespie tau-leaping method using graphics processing units. PLoS One 2012;7(6):e37370. 22715366 10.1371/journal.pone.0037370 PMC3371023

[107] Nobile MS, Cazzaniga P, Besozzi D, et al cuTauLeaping: A GPU-powered tau-leaping stochastic simulator for massive parallel analyses of biological systems. PLoS One 2014;9(3):e91963. 24663957 10.1371/journal.pone.0091963 PMC3963881

[108] Vigelius M, Lane A, Meyer B. Accelerating reaction-diffusion simulations with general-purpose graphics processing units. Bioinformatics 2011;27(2):288–90. 21062761 10.1093/bioinformatics/btq622

[109] Pasquale G, Maj C, Clematis A, et al A CUDA implementation of the Spatial TAU-leaping in Crowded Compartments (STAUCC) simulator In: 2014 22nd Euromicro International Conference on Parallel, Distributed and Network-Based Processing (PDP). Washington, DC: IEEE, 2014, 609–16.

[110] Gladkov DV, Alberts S, D’Souza RM, et al Accelerating the Smoldyn spatial stochastic biochemical reaction network simulator using GPUs In: Proceedings of the 19th High Performance Computing Symposia. Society for Computer Simulation International, 2011, 151–8.

[111] Dematté L. Smoldyn on graphics processing units: massively parallel Brownian dynamics simulations. IEEE/ACM Trans Comput Biol Bioinform 2012;9(3):655–67. 21788675 10.1109/TCBB.2011.106

[112] Richmond P, Walker D, Coakley S, et al High performance cellular level agent-based simulation with FLAME for the GPU. Brief Bioinform 2010;11(3):334–47. 20123941 10.1093/bib/bbp073

[113] D’Souza RM, Lysenko M, Marino S, et al Data-parallel algorithms for agent-based model simulation of tuberculosis on graphics processing units In: Proceedings of the 2009 Spring Simulation Multiconference. San Diego, CA: Society for Computer Simulation International, 2009.

[114] Cazzaniga P, Nobile MS, Besozzi D, et al Massive exploration of perturbed conditions of the blood coagulation cascade through GPU parallelization. BioMed Res Int 2014;2014:863298. 25025072 10.1155/2014/863298 PMC4082904

[115] Nobile MS, Harris L, Shockley E, et al GPU-powered sensitivity analysis of a large-scale model of death cell signaling and proliferation in cancer cells In: Proceedings 2015 Winter Q-Bio Meeting, Maui, HI, 2015.

[116] Nobile MS, Besozzi D, Cazzaniga P, et al A GPU-based multi-swarm PSO method for parameter estimation in stochastic biological systems exploiting discrete-time target series In: Giacobini M, Vanneschi L, Bush W (eds). Evolutionary Computation, Machine Learning and Data Mining in Bioinformatics. 10th European Conference, EvoBIO 2012. Proceedings, Vol. 7246 of Lecture Notes in Computer Science. Heidelberg, Berlin: Springer-Verlag, 2012, 74–85.

[117] Liepe J, Barnes C, Cule E, et al ABC-SysBio – approximate Bayesian computation in Python with GPU support. Bioinformatics 2010;26(14):1797–9. 20591907 10.1093/bioinformatics/btq278 PMC2894518

[118] Butcher JC. Numerical Methods for Ordinary Differential Equations. Chichester, West Sussex, England: John Wiley & Sons, 2003.

[119] The SBML portal. http://www.sbml.org/.

[120] Petzold LR. Automatic selection of methods for solving stiff and nonstiff systems of ordinary differential equations. SIAM J Sci Stat Comput 1983;4(1):136–48.

[121] Hoops S, Sahle S, Gauges R, et al COPASI—a COmplex PAthway SImulator. Bioinformatics 2006;22:3067–74. 17032683 10.1093/bioinformatics/btl485

[122] Chatterjee MS, Denney WS, Jing H, et al Systems biology of coagulation initiation: kinetics of thrombin generation in resting and activated human blood. PLoS Comput Biol 2010;6(9):e1000950. 20941387 10.1371/journal.pcbi.1000950 PMC2947981

[123] Gillespie DT. The chemical Langevin equation. J Chem Phys 2000;113:297–306.

[124] Gillespie DT. Exact stochastic simulation of coupled chemical reactions. J Comput Phys 1977;81(25):2340–61.

[125] Matsumoto M, Nishimura T. Mersenne twister: a 623-dimensionally equidistributed uniform pseudo-random number generator. ACM Trans Model Comput Simul 1998;8(1):3–30.

[126] Gillespie DT, Petzold LR. Improved leap-size selection for accelerated stochastic simulation. J Chem Phys 2003;119:8229–34.

[127] Cao Y, Gillespie DT, Petzold LR. Efficient step size selection for the tau-leaping simulation method. J Chem Phys 2006;124(4):044109. 16460151 10.1063/1.2159468

[128] Baras F, Malek MM. Reaction-diffusion master equation: a comparison with microscopic simulations. Phys Rev E 1996;54(6):6139–48. 10.1103/physreve.54.6139 9965833

[129] Hutchcraft EW, Maxwell W, Richard GK. On the acceleration of the numerical solution of partial differential equations using radial basis functions and graphics processing units. Int J Numer Model Electron Network Dev Field 2013;26(4):415–23.

[130] ElMaghrbay M, Ammar R, Rajasekaran S. Fast GPU algorithms for implementing the red-black Gauss-Seidel method for solving partial differential equations In: IEEE Symposium on Computers and Communications (ISCC), 2013. IEEE, 2013, 269–74.

[131] Minden V, Smith B, Knepley MG. Preliminary implementation of PETSc using GPUs In: Yuen DA, Wang L, Chi X, et al (eds). GPU Solutions to Multi-scale Problems in Science and Engineering, Lecture Notes in Earth System Sciences. Springer, 2013, 131–40.

[132] Mosca E, Cazzaniga P, Pescini D, et al Modelling spatial heterogeneity and macromolecular crowding with membrane systems In: Gheorghe M, Hinze T, Păun G, et al (eds). Membrane Computing, Vol. 6501 of Lecture Notes in Computer Science. Springer, 2011, 285–304.

[133] Andrews SS. Bacterial Molecular Networks: Methods and Protocols, chapter Spatial and Stochastic Cellular Modeling with the Smoldyn Simulator. New York, NY: Springer, 2012, 519–42. 10.1007/978-1-61779-361-5_26 22144170

[134] Christley S, Lee B, Dai X, et al Integrative multicellular biological modeling: a case study of 3D epidermal development using GPU algorithms. BMC Syst Biol 2010;4:107. 20696053 10.1186/1752-0509-4-107 PMC2936904

[135] Aldridge BB, Burke JM, Lauffenburger DA, et al Physicochemical modelling of cell signalling pathways. Nat Cell Biol 2006;8(11):1195–203. 17060902 10.1038/ncb1497

[136] Bartocci E, Lió P. Computational modeling, formal analysis, and tools for systems biology. PLoS Comput Biol 2016;12(1):e1004591. 26795950 10.1371/journal.pcbi.1004591 PMC4721667

[137] Miller J, Thomson P. Cartesian genetic programming In: Poli R, Banzhaf W, Langdon WB, et al (eds). Genetic Programming. European Conference, EuroGP 2000, Vol. 1802 of Lecture Notes in Computer Science. Springer-Verlag, 2000, 121–32.

[138] Johnson R, Kirk P, Stumpf MPH. SYSBIONS: Nested sampling for systems biology. Bioinformatics 2014;31(4):604–5. 25399028 10.1093/bioinformatics/btu675 PMC4325544

[139] Lomont C. Introduction to Intel Advanced Vector Extensions. Intel White Paper, 2011.

[140] Pennycook SJ, Hammond SD, Wright SA, et al An investigation of the performance portability of OpenCL. J Parallel Distr Com 2013;73(11):1439–50.

[141] Stone JE, Gohara D, Shi G. OpenCL: a parallel programming standard for heterogeneous computing systems. Comput Sci Eng 2010;12(1–3):66–73. 21037981 10.1109/MCSE.2010.69 PMC2964860

[142] Nvidia. NVRTC—CUDA runtime compilation 7.5, 2015 http://docs.nvidia.com/cuda/nvrtc/index.html.

[143] Harvey MJ, De Fabritiis G. Swan: a tool for porting CUDA programs to OpenCL. Comput Phys Commun 2011;182(4):1093–9.

[144] Nvidia. Nvidia CUDA C Programming Guide 7.5, 2015 http://docs.nvidia.com/cuda/cuda-c-programming-guide/.

[145] Nvidia. CUDA C Best Practices Guide 7.5, 2015 http://docs.nvidia.com/cuda/cuda-c-best-practices-guide/.

