# Supplementary material for: Graphics processing units in bioinformatics, computational biology and systems biology
Source: Brief Bioinform. 2016 Jul 7;18(5):870–85. doi: 10.1093/bib/bbw058 (PMC5862309; doi:10.1093/bib/bbw058)
Supplement: Supplementary Data [file bbw058_supplfile_1.pdf]

Supplementary File 1

Additional GPU-powered Tools for Bioinformatics and Computational Biology

Marco S. Nobile, P. Cazzaniga, A. Tangherloni, D. Besozzi

This document presents a collection of additional GPU-powered tools developed for Bioinformatics, Computational Biology and Systems Biology. All tools mentioned hereby are listed in Table 1.

Spectral Analysis

A field of Computational Biology where GPU acceleration can yield a relevant speed-up is related to the analysis of spectral data derived from, e.g., mass-spectrometry experiments.

FastPaSS [4] is a tool that accelerates the identification of a spectrum in a spectral library by means of the SpectraST similarity scoring algorithm [14]. The core of the matching algorithm is represented by a dot product of two vectors corresponding to the normalized intensities of the spectra, a calculation that is well-suitable for GPU acceleration. According to the results shown in [4], using a Nvidia GeForce 8600 GTS, FastPaSS allows a 8× speed-up with respect to a sequential execution optimized using a pre-caching of files.

Tempest [19] extends the task of spectral matching by performing the whole similarity scoring—including the generation of theoretical fragmented spectra—directly on the GPU. Tempest exploits the possibility of asynchronous execution of CUDA kernels to implement a heterogeneous execution scheme, in which the CPU performs database digestion while the GPU performs the scoring. Tempest implements two scoring functions: the first based on cross-correlation, and the second on dot product. According to [19], Tempest's accelerated scoring function based on the dot product allows a two orders of magnitude speed-up with respect to the correlation-based method. Still, the approach based on correlation is more accurate and Tempest's speed-up is about 10× with respect to the CPU, using a Nvidia GeForce GTX 480.

The acceleration of similarity scoring by means of GPUs was also investigated in [16]. Here, it was shown that the spectral dot product can be strongly accelerated by intensively exploiting the shared memory and parallel reduction techniques, with a two-order magnitude speed-up using a Nvidia GeForce GTX 280.

A different example of GPU-powered spectral analysis concerns feature detection, used to identify and quantify the proteins contained in a sample; in particular, features must be separated from signal noise and baseline artifacts. Hussong *et al.* [11] employed an adaptive wavelet transform for this task, a process that can be parallelized on the GPU. Their implementation exploits the shared memory to store the spectrum, allowing a 200× speed-up on real world data sets. Since the methodology was implemented on an early CUDA architecture (namely, Nvidia Tesla C870), the amount of available memory was limited to 16 KB (see Table 2 in Supplementary File 2). Thus, for larger signals, this tool automatically switches to texture memory, affecting the speed-up.

Genome-wide Analysis

The aim of genome-wide analysis is the investigation of cellular processes at large scale, trying to encompass the complexity of whole cells. One approach for genome-wide analysis consists in the extraction of information from a given interaction network, formalized as a graph whose vertexes represent the biochemical elements, and edges between vertexes correspond either to a physical/functional interaction

or to some kind of correlation. These networks can be analyzed from a topological perspective, in order to capture some information on the network structure [3].

In this context, FastGCN exploits GPUs for building interaction networks of co-expressed genes [17]. Interestingly, despite an intense optimization including branch removal from source code, compressed data structures and coalesced access patterns, the GPU version of FastGCN can be slower than a multi-threaded CPU implementation when analyzing small datasets. However, according to the results presented in [17], FastGCN is 63× faster than a single-thread version implemented using the R language.

The goal of genome-wide association studies (GWAS) is to determine the genetic variations of a population that are responsible for a specific phenotype. Since this methodology relies on genome-wide information, it is in general computationally challenging. To this aim, GBOOST [25] was developed to perform gene-gene interaction analysis of large genome data, showing a 40× speed-up and therefore reducing the running time of a GWAS from 2.5 days down to a few hours.

PBOOST [24] is another tool developed in the context of GWAS, which performs a permutation test to detect interacting SNP pairs having significant association with diseases. In order to assess a meaningful P-value, a huge number of permutations must be tested, a circumstance that motivated the GPU implementation; thanks to GPU acceleration, PBOOST is able to strongly reduce the running time. The authors analyzed 10^7 permutations for a single SNP pair considering the genomic data from the Wellcome Trust Case Control Consortium [5]: the analysis was completed in 1 minute using a GPU Nvidia Tesla M2090, instead of the 60 minutes required by a CPU Intel Xeon E5-2650.

GWAS also relies on haplotyping and imputation of untyped genotypes, two tasks whose computational cost escalates quadratically with the number of reference haplotypes. Mendel-GPU [8] performs accelerated imputation, reducing the running time from around 3 years down to less than a week. Finally, PANET [23] is a GPU-powered tool created to investigate how feedback and feedforward loops determine the robustness of the dynamics of large-scale biological networks. PANET was designed to overcome the limitations of a previous software (namely, NetDS [15]), whose applicability to genome-wide networks was limited by CPU-bound execution. To the best of our knowledge, PANET and Mendel-GPU are the only tools for computational analysis of biological systems implemented exclusively for the OpenCL framework.

Bayesian Inference

Three notable examples of GPU-based methodologies for Bioinformatics are both based on Bayesian inference: MrBayes [2], PLL [12] and FamSeq [21]. The first two tools are used for the investigation of phylogenetic trees from DNA data, while the third calculates the variant calling for family-based sequencing data.

The GPU version of MrBayes achieved a 63× speed-up with respect to the equivalent CPU-bound implementation, although it is characterized by a limited applicability [12]. The authors also tested a distributed and multi-GPU execution on the Tianhe-1A supercomputer, using 32 of its 7168 GPU Nvidia M2050, obtaining a noticeable 478× acceleration. A further version of MrBayes, named oMC^3, was proposed by Chai *et al.* [7]: this heterogeneous implementation further reduced the running time by simultaneously exploiting multi-threaded CPU computation and GPUs.

The authors of PLL compared the performance of a GPU implementation (running on a Nvidia Tesla C2075) with a strongly optimized CPU version exploiting AVX intrinsics [18], with a maximum speed-up of 2×.

In the case of FamSeq, the GPU was exploited to calculate the posterior probability for 3^n kinds of genotypes, where n is the pedigree size: this is a task suitable for GPU's programming paradigm. Thanks to this strategy, the tool achieved a 10× speed-up that, as stated by the authors, allows to call variants for the whole genome sequencing data in just 36 hours, instead of 16 days as required by the CPU version.

Movement Tracking

Movement tracking algorithms can be used to assist model building and validation. Szafaryn *et al.* [22] tested a MATLAB application for heart wall tracking, which exploits external CUDA kernels to offload

highly parallel activities (e.g., speckle-reducing anisotropic diffusion). According to their results, GPUs allow a relevant speed-up; however, the authors underline the difficulty in porting existing algorithms to GPUs despite their advantages, explicitly stating that the improvement of performances is directly proportional to the coding effort.

Quantum Chemistry

Quantum chemistry is based on computationally demanding simulation methods that rely on models of the electronic structure of many-body systems, and exploit approximate solutions of the Schrödinger equation [13]. Different CUDA implementations of these methods—based on Khon-Sham and Hartree-Fock theories, *ab initio* electron correlation techniques and quantum Monte Carlo—achieved up to 100× speed-up with respect to the classic sequential counterpart. For these topics, we refer the interested reader to [10] and references therein.

Further General Techniques

Additional applications of GPUs can be exploited to accelerate the investigation of complex biological systems. For instance, Nvidia's cuFFT libraries allow the accelerated calculation of the Fast Fourier Transform [20]; Principal Components Analysis, widely used to reduce the complexity of biological systems, can be accelerated on GPUs [1]; Markov Clustering, which can be exploited to identify functional modules in protein-protein interaction networks, is well suited for GPU's acceleration, provided that the implementation exploits a sparse matrix data structure [6]; the Fast Non-dominating Sorting Genetic Algorithm (NSGA-II) is useful to solve many multi-objective optimization tasks in Computational and Systems Biology [9].

**Miscellaneous**				
	**Tool name**	**Speed-up**	**Parallel solution**	**Reference**
Identification of spectra in spectral analysis	FastPaSS	8×	GPU	[4]
Identification of spectra in spectral analysis	Tempest	10×	GPU	[19]
Identification of spectra in spectral analysis	-	100×	GPU	[16]
Spectral analysis with wavelet transform	-	200×	GPU	[11]
Genome-wide interaction networks of co-expressed genes	FastGCN	63×	GPU	[17]
Genome-wide gene-gene interaction analysis	GBOOST	40×	GPU	[25]
Interacting SNP pairs detection	PBOOST	60×	GPU	[24]
Haplotyping	Mendel-GPU	-	GPU	[8]
Large-scale network robustness analysis	PANET	-	GPU	[23]
Phylogenetic trees from DNA data	MrBayes	63× (478×*)	GPU	[2]
Phylogenetic Likelihood Library for DNA data	PLL	2×	GPU	[12]
Family-based sequencing data	FamSeq	10×	GPU	[21]
Phylogenetic trees from DNA data	oMC^3	-	CPU-GPU	[7]
Heart wall tracking	-	-	CPU-GPU	[22]
Quantum chemistry	-	100×	GPU	[10]
Multi-objective optimization with Genetic Algorithm	NSGA-II	$10\times - 130\times$	GPU	[9]

Table 1: List of the GPU-powered miscellaneous tools, along with the speed-up achieved and the solutions used for code parallelization.
*Refers to a speed-up value achieved on a cluster of GPUs.

References

[1] M. Andrecut. Parallel GPU implementation of iterative PCA algorithms. *J Comput Biol*, 16(11):1593–1599, 2009.

[2] J. Bao, H. Xia, J. Zhou, et al. Efficient implementation of MrBayes on multi-GPU. *Mol Biol Evol*, 30(6):1471–1479, 2013.

[3] A.-L. Barabási and Z. N. Oltvai. Network biology: understanding the cell's functional organization. *Nat Rev Genet*, 5(2):101–113, 2004.

[4] L. A. Baumgardner, A. K. Shanmugam, H. Lam, et al. Fast parallel tandem mass spectral library searching using GPU hardware acceleration. *J Proteome Res*, 10(6):2882–2888, 2011.

[5] P. R. Burton, D. G. Clayton, L. R. Cardon, et al. Genome-wide association study of 14,000 cases of seven common diseases and 3,000 shared controls. *Nature*, 447(7145):661–678, 2007.

[6] A. Bustamam, K. Burrage, and N. A. Hamilton. Fast parallel Markov clustering in bioinformatics using massively parallel computing on GPU with CUDA and ELLPACK-R sparse format. *IEEE ACM T Comput Biol*, 9(3):679–692, 2012.

[7] J. Chai, H. Su, M. Wen, et al. Resource-efficient utilization of CPU/GPU-based heterogeneous supercomputers for Bayesian phylogenetic inference. *J Supercomput*, 66(1):364–380, 2013.

[8] G. K. Chen, K. Wang, A. H. Stram, et al. Mendel-GPU: haplotyping and genotype imputation on graphics processing units. *Bioinformatics*, 28(22):2979–2980, 2012.

[9] D. D'Agostino, G. Pasquale, and I. Merelli. A fine-grained CUDA implementation of the multi-objective evolutionary approach NSGA-II: Potential impact for Computational and Systems Biology applications. In C. Di Serio, P. Liò, A. Nonis, and R. Tagliaferri, editors, *Computational Intelligence Methods for Bioinformatics and Biostatistics*, volume 8623 of *Lecture Notes in Computer Science*, pages 273–284. Springer, 2015.

[10] A. W. Götz, T. Wölfle, and R. C. Walker. Quantum chemistry on graphics processing units. *Annu Rep Comput Chem*, 6:21–35, 2010.

[11] R. Hussong, B. Gregorius, A. Tholey, et al. Highly accelerated feature detection in proteomics data sets using modern graphics processing units. *Bioinformatics*, 25(15):1937–1943, 2009.

[12] F. Izquierdo-Carrasco, N. Alachiotis, S. Berger, et al. A generic vectorization scheme and a GPU kernel for the Phylogenetic Likelihood Library. In *Parallel and Distributed Processing Symposium Workshops & PhD Forum (IPDPSW), 2013 IEEE 27th International*, pages 530–538. IEEE, 2013.

[13] W. Kohn and L. J. Sham. Self-consistent equations including exchange and correlation effects. *Phys Rev D*, 140(4A):A1133, 1965.

[14] H. Lam, E. W. Deutsch, J. S. Eddes, et al. Development and validation of a spectral library searching method for peptide identification from MS/MS. *Proteomics*, 7(5):655–667, 2007.

[15] D. H. Le and Y. K. Kwon. NetDS: a Cytoscape plugin to analyze the robustness of dynamics and feedforward/feedback loop structures of biological networks. *Bioinformatics*, 27(19):2767–2768, 2011.

[16] Y. Li and X. Chu. Speeding up scoring module of mass spectrometry based protein identification by GPU. In *High Performance Computing and Communication & 2012 IEEE 9th International Conference on Embedded Software and Systems (HPCC-ICESS), 2012 IEEE 14th International Conference on*, pages 1315–1320. IEEE, 2012.

[17] M. Liang, F. Zhang, G. Jin, et al. FastGCN: A GPU accelerated tool for fast gene co-expression networks. *PLoS ONE*, 10(1):e0116776, 2014.

[18] C. Lomont. Introduction to Intel Advanced Vector Extensions. *Intel White Paper*, 2011.

[19] J. A. Milloy, B. K. Faherty, and S. A. Gerber. Tempest: GPU-CPU computing for high-throughput database spectral matching. *J Proteome Res*, 11(7):3581–3591, 2012.

[20] Nvidia. cuFFT library user's guide 7.5, February 2015.

[21] G. Peng, Y. Fan, and W. Wang. FamSeq: A variant calling program for family-based sequencing data using graphics processing units. *PLoS Comput Biol*, 10(10):e1003880, 2014.

[22] L. G. Szafaryn, K. Skadron, and J. J. Saucerman. Experiences accelerating MATLAB systems biology applications. In *Proceedings of the Workshop on Biomedicine in Computing: Systems, Architectures, and Circuits*, pages 1–4, 2009.

[23] H. C. Trinh, D. H. Le, and Y. K. Kwon. PANET: A GPU-based tool for fast parallel analysis of robustness dynamics and feed-forward/feedback loop structures in large-scale biological networks. *PLoS ONE*, 9(7):e103010, 2014.

[24] G. Yang, W. Jiang, Q. Yang, et al. PBOOST: A GPU based tool for parallel permutation tests in genome-wide association studies. *Bioinformatics*, 31(9):1460–1462, 2014.

[25] L. S. Yung, C. Yang, X. Wan, et al. GBOOST: a GPU-based tool for detecting gene-gene interactions in genome-wide case control studies. *Bioinformatics*, 27(9):1309–1310, 2011.
