# Supplementary material for: Graphics processing units in bioinformatics, computational biology and systems biology
Source: Brief Bioinform. 2016 Jul 7;18(5):870–85. doi: 10.1093/bib/bbw058 (PMC5862309; doi:10.1093/bib/bbw058)
Supplement: Supplementary Data [file bbw058_supplfile_2.pdf]

Supplementary File 2
Graphics Processing Units and CUDA

M. S. Nobile, P. Cazzaniga, A. Tangherloni, D. Besozzi

The Advantages of Graphics Processing Units

In the context of high-performance computing (HPC), the traditional solutions for distributed architectures are represented by computer clusters and grid computing [10]. The former exploits a set of inter-connected computers controlled by a centralized scheduler, while the latter consists in the logical organization of a set of geographically distributed (heterogeneous) computing resources. In both cases, the overall computational task is partitioned into smaller sub-tasks, which are assigned to the various computing units for parallel or distributed computation. These infrastructures are particularly appealing because they usually require minimal changes to the existing source code of some given program: as a matter of fact, the computing units are generally based on classic architectures (e.g., the x86 instruction set, typical of personal computers), so that the code can be easily ported, with the exception of possible modifications required for message passing. Moreover, both architectures support the *Multiple Instruction Multiple Data* (MIMD) execution paradigm, that is, all computing units are independent, asynchronous, can work on different data and execute different code.

Despite these advantages, computer clusters and grid computing have considerable drawbacks. On the one hand, computer clusters are expensive, require maintenance and are characterized by relevant energy consumption. On the other hand, grid computing [8] is generally based on *volunteering*, whereby computer owners donate resources (e.g., computing power, storage) to a specific project [2, 3]. Several factors may further affect grid computing, notably the fact that remote computers might not be completely trustworthy: potentially unpublished data are transmitted to unknown remote clients for processing, and returned results might be intentionally erroneous or misleading. Moreover, there is no general guarantee about the availability of remote computers, so that some allocated tasks could never be processed.

A third way to distributed computation is the emergent field of cloud computing, in which private companies offer a pool of computation resources (e.g., computers, storage) attainable on-demand and ubiquitously over the Internet. Although cloud computing mitigates some problems of classic distributed architectures—like the costs of the infrastructure and its maintenance—it is characterized by other problems, mainly the fact that data are stored on servers owned by private companies. This brings about issues of privacy, potential piracy, espionage, continuity of the service (e.g., due to some malfunctioning, DDoS attacks, or Internet connection problems), international legal conflicts, *data lock-in*, along with typical problems of Big Data, e.g., transferring terabyte-scale data to and from the cloud [4].

In the latter years, a completely different approach to HPC gained ground: the use of general-purpose multi-core devices like Many Integrated Cores (MIC) co-processors [23] and Graphics Processing Units (GPUs) [18]. Noteworthy, both types of devices can be installed on common consumer computers and are characterized by a large number of computing cores (up to 61 for MICs and 5760 for GPUs, at the time of writing).

MICs are characterized by cores based on the x86 instruction set, extended with 512-bit vectorial instructions, inter-connected by means of a ring bus. Thanks to this architectural choice, any existing code developed for Central Processing Units (CPUs) should be easily ported to the MIC architecture. In addition, MICs offer two main programming models: the *native model*, which allows the execution of the source code directly on the MIC, exploiting the multiple cores for parallel execution, and the heterogeneous *offload model*, which allows to use simple compiler directives to designate the code sections that are executed on the MIC, while the rest of the code runs on the CPU.

Differently from MICs, GPUs are pervasive, relatively cheap and extremely efficient parallel multi-core

co-processors, that were originally designed to accelerate the real-time rendering of computer graphics, freeing the CPU for further calculations. Nowadays, even common consumer machines are equipped with GPUs, whose exceptional computing power can be exploited to obtain, with a single machine, the same performances of clusters and grid, without the need for job scheduling or the transfer of confidential information. This HPC methodology, named *general-purpose GPU* (GPGPU) *computing*, is a recent paradigm that gives access to low-cost, energy-efficient means to achieve tera-scale performances on common workstations (and peta-scale performances on GPU-equipped supercomputers [11, 5]), obtained by leveraging the powerful parallel capabilities of modern video cards.

Despite the relevant performance-per-watt and performance-per-price ratio, also GPGPU computing has some drawbacks. The first is related to the fact that GPUs are mainly designed to provide the *Same Instruction Multiple Data* (SIMD) parallelism. That is, differently from the MIMD architecture, all cores in the GPU are supposed to execute the same instructions on different input data: this is not the usual execution strategy for existing CPU implementations, therefore the CPU code cannot be directly ported to the GPU's architecture. In general, the code needs to be rewritten for GPUs, which are completely different architectures and support a different set of functionalities, as well as different libraries. In addition, the complex hierarchy of memories and the limited amount of high-performance memories available on GPUs require a redesign of the existing algorithms, to better fit and fully leverage this architecture. Thus, from the point of view of the software developer, GPU programming remains a challenging task [7]. Nevertheless, GPGPU computing represents a valuable and even "green" alternative to traditional HPC infrastructures: indeed, GPUs offer the possibility of creating supercomputers that are orders of magnitude faster than conventional clusters [11, 5], but having a comparable energy consumption. Empirical comparisons of equivalent implementations have also shown that GPUs attain better performances than MICs [6, 22], especially when the code is not purposely modified to leverage the additional MICs throughput allowed by vectorial instructions. For these reasons, although GPUs require a relevant programming effort, they still represent one of the most attractive alternatives for biology-oriented HPC.

We also mention that a further option for HPC is the use of reconfigurable hardware platforms such as Field Programmable Gates Arrays (FPGAs) [21]. However, they require dedicated hardware, very specific programming skills for circuits design, and yield worse performances than multi-core devices.

The SIMD paradigm, used by GPUs, dates back to the original purpose of accelerating real-time three-dimensional computer graphics [12]. Thus, modern multi-core GPUs can process, in parallel, a massive number of geometric primitives in a multi-threaded and pipelined fashion. To this aim, GPUs exploit a large number of cores to speedup the parallel processing of such primitives. To facilitate the development of scientific code on GPUs, novel Application Programming Interface (APIs) tailored for general-purpose computation were lately introduced: CUDA (created by Nvidia, and compatible only with Nvidia GPUs), OpenCL (proposed by the Khronos Group, compatible with any GPU) and Microsoft DirectCompute (compatible with Microsoft Windows operating system and any GPU).

Among the existing libraries for GPGPU computing, CUDA is by far the most used in the fields of Bioinformatics, Computational Biology and Systems Biology, representing the *standard de facto* in the field of scientific computation; a detailed overview of this library is provided in the next section.

Compute Unified Device Architecture

In order to provide a simple library for GPGPU computing, Nvidia developed a new parallel computing platform and programming model named CUDA (Compute Unified Device Architecture), programmable using the C language and based on multi-core *Streaming Multiprocessors* (SMs). The CUDA compiler `nvcc` subdivides this "extended" C code into two separate parts: the host (i.e., the CPU) code and the device (i.e., the GPU) code. The former is compiled using any C++ compiler that is available on the machine, while the second is processed using a proprietary Nvidia compiler. The device code is then embedded as binary image into the host object file. Finally, during the linking stage, CUDA runtime libraries are added to the binary executable file.

The compilation of device code is performed using an intermediate stage during which a special representation, namely PTX, is used. PTX can be seen as the equivalent of assembly code for a "virtual GPU", being an Instruction Set Architecture that is supposed to remain stable and uniform across multiple generations of GPUs. The PTX is translated for one (or more) specific target architectures,

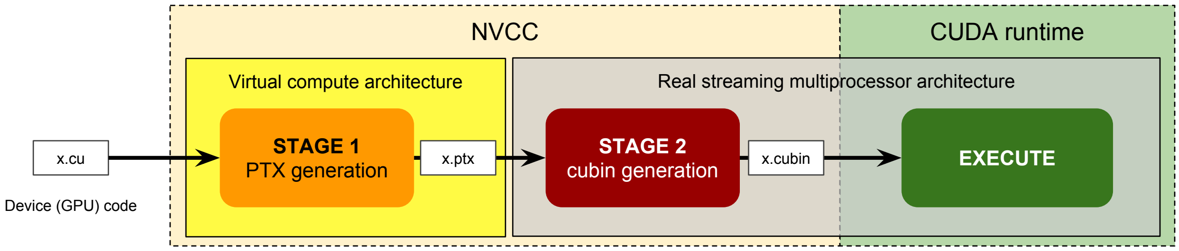

Figure 1: Two-staged compilation with virtual and real architectures. Figure adapted from [13].

supporting the capabilities of this virtual GPU. A schematization of this process is reported in Figure 1. It is worth noting that this process can be performed automatically by `nvcc`, or it can be split into two separate phases. In the latter case, the intermediate PTX files can be modified before compilation, in order to drive a desired behavior or to leverage special device instructions that might not be directly supported by CUDA C (e.g., SIMD instructions).

CUDA is nowadays a mature architecture and offers several additional libraries that make GPGPU computing easier for developers, for instance CURAND [16] (which provides high-quality pseudo- and quasi-random numbers generators), cuFFT [15] (a Fast Fourier Transform library) or Thrust [9] (a C++ template library for CUDA based on the Standard Template Library). A list of the most common CUDA libraries and the functionalities they provide is reported in Table 1.

Table 1: Main libraries available for CUDA.

Name of the library	*Purpose of the library*	*Reference*
cuBLAS	Linear algebra	[14]
cuFFT	Fast Fourier transform computation	[15]
cuRAND	Random numbers generation	[16]
cuSPARSE	Linear algebra subroutines for sparse matrices	[17]
NPP	Primitives for image and signal processing	[19]
NVBIO	High-throughput sequence analysis	[1]
Thrust	Library of common data structures and parallel algorithms (e.g., sort, scan, transform, and reduction operations)	[9]

CUDA code can run on the most widespread operating systems (Microsoft Windows, Apple OSX, GNU/Linux), although it requires a Nvidia GPU. More precisely, CUDA code can be compiled but cannot be executed on host machines equipped with ATI/AMD or Intel video cards: in order to exploit GPUs made by these vendors, alternative libraries (e.g., OpenCL) must be used. Each new generation of Nvidia GPUs offers novel or improved characteristics; the subset of features supported by a specific hardware determines its *Compute Capability* (CC). Table 2 reports an overview of the various CUDA architectures to date, along with their CCs and some of the novel functionalities they introduced.

CUDA can be programmed by means of C/C++ language: following CUDA's naming conventions, the developer implements a C/C++ function (called *kernel*) which is loaded from the host (the CPU) to the devices (one or more GPUs), and replicated in many copies named *threads*. Threads are organized in three-dimensional structures named *blocks*, contained in three-dimensional *grids* (see Figure 2). Whenever the host computer runs a kernel, the GPU creates the corresponding grid and automatically schedules each block of threads on an available SM, allowing a transparent scaling of performances on different devices (see Figure 3).

During the launch of a kernel, CUDA requires the programmer to specify how many threads T are assigned to each block, and how many blocks B must be created. The total number of running threads on the GPU will be equal to $T \times B$. CUDA poses limitations to the number of threads that a single

Table 2: Some relevant architectural innovations and new features introduced by progressive compute capabilities in different CUDA architectures. CUDA architectures and compute capabilities are ordered from the oldest to the latest. For each architecture, the most recent compute capability generally maintains or improves the functionalities provided by the previous compute capabilities.

CUDA architecture	*Compute capability*	*Architectural innovations and new features*
Tesla	1.0	Up to 512 threads per block and 65536 blocks per grid, up to 16 KB of shared memory per SM, up to 8 simultaneous blocks per SM, up to 63 registers per thread
	1.1	Atomic functions in global memory
	1.2	Atomic functions in shared memory and warp voting
	1.3	Double precision floating point operations
Fermi	2.0	Up to 1024 threads per block, up to 48 KB of shared memory per SM, L2 cache on global memory, custom balancing of L1 cache and shared memory (48+16 KB vs 16+48 KB)
	2.1	Advanced synchronization functions
Kepler	3.0	Unified memory programming, up to $2^{31}-1$ blocks per grid, up to 16 simultaneous blocks per SM, up to 255 registers per thread, balanced L1 cache/shared memory configuration (32+32 KB)
	3.5	Dynamic parallelism
Maxwell	5.0	Up to 64 KB of shared memory per SM, up to 32 blocks per SM
	5.2	Up to 96 KB of shared memory per SM
Pascal	6.0	Base clock up to 1480 MHz with GPU Boost, 4 MB of L2 cache, 4096-bit high-performance HBM2 memories

block can contain. For instance, T was limited to 512 on Tesla GPUs made by Nvidia, while it is limited to 1024 on more recent architectures (see Table 2). Within a block, threads can be uniformly distributed by the user along the three dimensions, given that x- and y-dimensions not exceed 1024 threads, while z-dimension must be limited to 64 threads. For instance, Figure 2 shows an example of three-dimensional blocks, each consisting of $3 \times 3 \times 3 = 27$ threads. The number of simultaneous threads on a SM is further limited by the available physical resources (e.g., registers, high performance memories). It follows that the performances of CUDA kernels are tied to the choice of the values T and B.

The CUDA programming model combines SIMD and multi-threading: the programmer launches a large number of identical threads that are supposed to perform identical computations on different data; however, threads are allowed to temporarily take divergent control flows (e.g., when the code contains `if-then-else` constructs). Nvidia refers to this peculiar architecture as SIMT: *Single Instruction Multiple Threads*. Figure 4 provides an example of a divergent execution flow, in which 16 threads are executing a common CUDA kernel (shown in the gray box on the right). The CUDA kernel is characterized by two nested conditional branches: a first branch is taken by threads whose variable x is greater than zero (yellow region); the second branch is taken by the remaining threads, whose variable y is greater than 0 (green region); the last branch is taken by the remaining threads (purple region). These three regions cannot be simultaneously executed: CUDA automatically handles the workflow by serializing the execution. Serialization affects performances, since it implies an increased running time. For this reason, conditional branches should be avoided in the kernels as much as possible. In the example shown in Figure 4, the last conditional branch (taken by threads which satisfy $x \leq 0$ and $y \leq 0$) causes the execution of a single thread at once (thread #10). It is clear that, in order to fully leverage the GPU, algorithms might require a major redesign to reduce the recourse to conditional branches.

GPUs are equipped with a variety of memories. As schematized in Figure 5, the GPU memory hierarchy consists in the *global memory* (accessible from all threads), the *shared memory* (accessible from threads belonging to the same block), the *registers* (high-performance on-chip memories used to

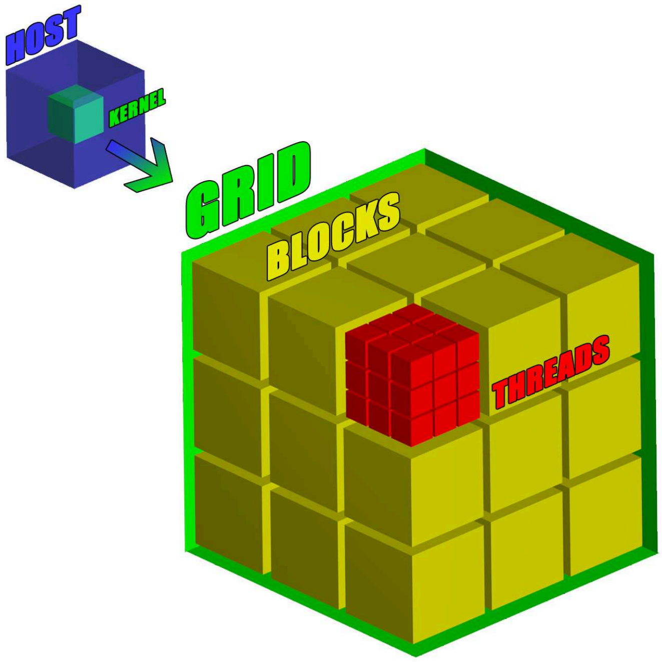

Figure 2: CUDA threads organization: a kernel (represented by the light blue cube), invoked from the host (blue cube), is executed in multiple threads on the GPU (the device). Threads (red cubes) are organized in three-dimensional structures named blocks. Blocks (yellow cubes) are organized in a three-dimensional structure named grid (green cube). During the kernel launch, the programmer must specify the dimensions of both blocks and grid. Thanks to this three-dimensional arrangement, threads in a block and blocks in a grid are addressed by a triple of coordinates: the darker red thread in the figure, for instance, belongs to block (0,0,0) and, inside this block, it is identified by coordinates (1,0,0), being the second thread along the x axis and the first along the y and z axes.

store local variables), the *local memory* (part of the global memory, accessible exclusively by the owner thread), and the *constant memory* (cached and not modifiable). One additional type of CUDA memory is the *texture memory*, which is another cached and read-only memory. Formerly designed to speedup the texture mapping in real-time 3D graphics, the texture memory was later exploited by CUDA to provide cached access to non modifiable data. However, since the introduction of the Fermi architecture, the global memory is equipped with a cache system (see Table 2) so that the texture memory is no longer useful, except for its hardware-accelerated trilinear interpolation capabilities. Nvidia increased the L2 cache to 4 MB on the most recent architecture, namely Pascal [20], at the time of writing. This increment of high-performance cache reduces the number of requests to the DRAM, improving the overall performances even on older softwares.

The best performances in the execution of CUDA code can be achieved by smartly distributing the data structures across these memories and, in particular, by exploiting the shared memory as much as possible. Unfortunately, the latter is a very limited resource (i.e., 96 KB for each multi-processor on the recent Maxwell GPUs, see Table 2), causing further restrictions on blocks' size. On the contrary, the global memory is very large (some GB in the most recent GPUs) but suffers of high latencies, a problem mitigated by the introduction of the aforementioned caching system. Also registers may pose a limitation to performances, since they are a scarce resource. When the number of registers required by a kernel outnumbers the available physical memories, the so-called *register spilling* on global memory occurs: the local variables are stored into the (slow) global memory, affecting the overall performances. Initially, GPUs were equipped with just 63 registers per thread; with the introduction of GPUs Kepler with compute capability 3.5, the number of threads was increased to 255 (see Table 2), reducing the recourse to the global memory and directly improving the performances in the case of complex kernels.

Finally, three additional matters concerning GPUs and their programming are worth to be mentioned. The first is that, since blocks are asynchronously scheduled on different SMs, there exist no

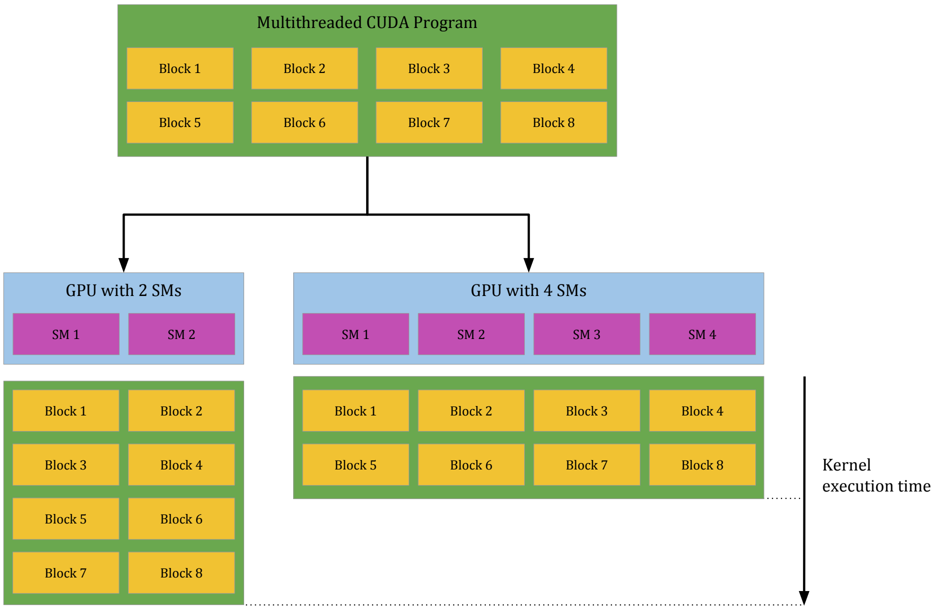

Figure 3: CUDA automatic scalability: blocks are automatically scheduled on the available SMs, in a transparent way for the programmer. The more SMs are present on the GPU, the faster the execution of the kernel.

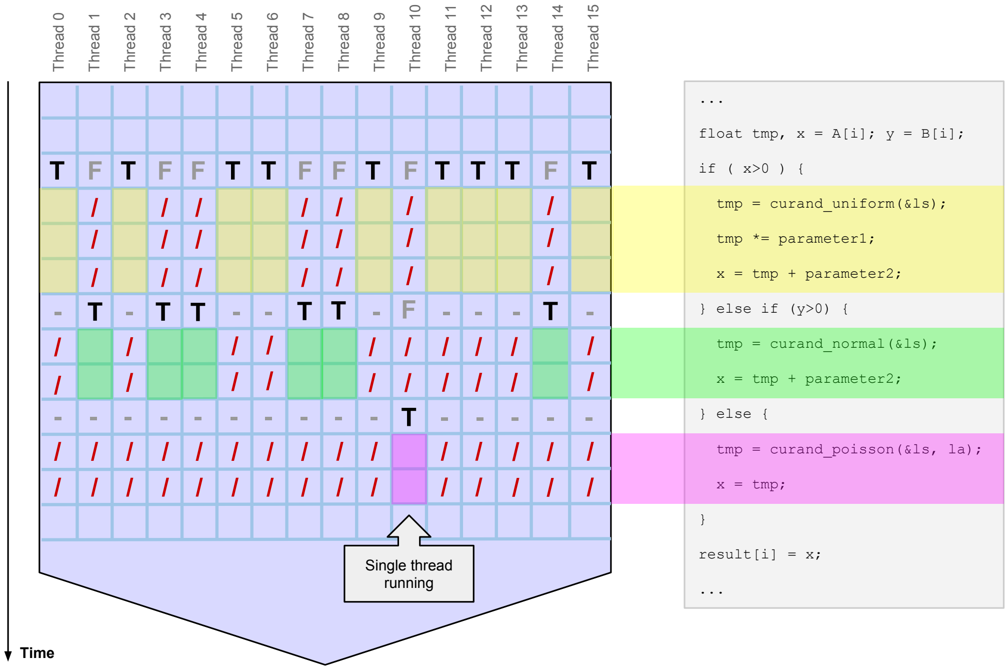

Figure 4: A simple example of threads divergence. Threads are grouped according to the conditional branches, which are evaluated at run-time. Then, threads performing the same calculations are executed together (e.g., the yellow parts). In the worst case scenario, the conditional branches can cause a complete serialization of the execution (e.g., the purple parts), hence reducing parallelism and strongly affecting the overall performances.

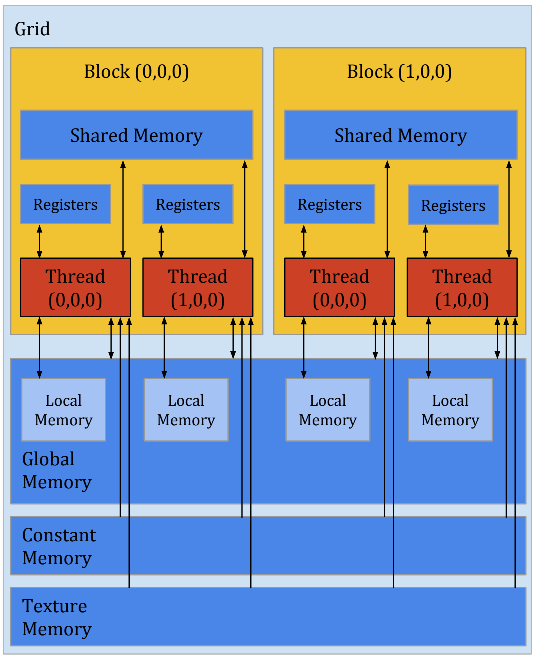

Figure 5: Architecture of CUDA's threads and memories hierarchy. Threads can access data from multiple kinds of memories, all with different scopes and characteristics: registers and local memories are private for each thread; shared memory let threads belonging to the same block communicate, and has low access latency; all threads can access the global memory, which suffers of high latencies, but it is cached since the introduction of the Fermi architecture; texture and constant memory can be read from any thread and are equipped with a cache as well.

communication nor synchronization primitives between blocks. However, intra-block communication can be performed by means of the shared memory and the family of *warp vote* functions, which allow the evaluation of a predicate for all threads and the recombination of the results. Intra-block synchronization is achieved using the family of `__syncthreads` functions, which can be also combined with warp voting. Some GPU-powered tools discussed in the main text of the review relied on this characteristics to leverage the peculiar architecture of GPUs.

The second is that, during the execution of a kernel, the CPU is idle and is allowed to execute additional useful tasks on the host side. This approach, which permits to fully leverage the computing power of a GPU-powered machine, is called *heterogeneous* computing. A different approach for leveraging the CPU during the execution of a kernel is the asynchronous data transfer: CUDA allows the creation of multiple *streams*, i.e., execution queues which handle the concurrent execution of tasks, including overlapped kernel runs and memory transfers. Some implementations discussed in the main text of the review exploited this feature to improve the performances.

Finally, the third is that global memory accesses can be optimized by the CUDA driver as long as the way data are organized—i.e., the access pattern—is *coalesced*, that is, the memory areas read by threads in a warp are contiguous. Although this complication has been mitigated by the most recent GPU architectures, a proper organization of data into coalesced patterns is necessary to reduce the number of memory transactions and improve the performances.

References

[1] NVBIO webpage. `http://nvlabs.github.io/nvbio/`.

[2] D. P. Anderson. BOINC: A system for public-resource computing and storage. In *Grid Computing, 2004. Proceedings. Fifth IEEE/ACM International Workshop on*, pages 4–10. IEEE, 2004.

[3] D. P. Anderson and G. Fedak. The computational and storage potential of volunteer computing. In *Cluster Computing and the Grid, 2006. CCGRID 06. Sixth IEEE International Symposium on*, volume 1, pages 73–80. IEEE, 2006.

[4] M. Armbrust, A. Fox, R. Griffith, et al. A view of cloud computing. *Comm ACM*, 53(4):50–58, 2010.

[5] A. S. Bland, J. C. Wells, O. E. Messer, et al. Titan: Early experience with the Cray XK6 at Oak Ridge National Laboratory. In *Proceedings of Cray User Group Conference (CUG 2012)*, 2012.

[6] P. Cazzaniga, F. Ferrara, M. S. Nobile, et al. Parallelizing biochemical stochastic simulations: a comparison of GPUs and Intel Xeon Phi processors. In V. Malyshkin, editor, *13th International Conference on Parallel Computing Technologies, PaCT 2015. Proceedings*, volume 9251 of *Lecture Notes in Computer Science*, pages 363–374, 2015.

[7] R. M. Farber. Topical perspective on massive threading and parallelism. *J Mol Graphics Model*, 30:82–89, 2011.

[8] I. Foster and C. Kesselman. *The Grid 2: Blueprint for a New Computing Infrastructure*. Elsevier, 2003.

[9] J. Hoberock and N. Bell. Thrust: A parallel template library, 2010. Version 1.7.0.

[10] A. Iosup and D. Epema. Grid computing workloads. *Internet Comput, IEEE*, 15(2):19–26, 2011.

[11] W. Joubert, R. Archibald, M. Berrill, et al. Accelerated application development: The ORNL Titan experience. *Comput Electr Eng*, 46:123–138, 2015.

[12] J. Nickolls and W. J. Dally. The GPU computing era. *IEEE Micro*, 30(2):56–69, 2010.

[13] Nvidia. Nvidia CUDA C Programming Guide v5.0, 2012.

[14] Nvidia. cuBLAS library 7.5, May 2015.

[15] Nvidia. cuFFT library user's guide 7.5, February 2015.

[16] Nvidia. CURAND library programming guide 7.5, February 2015.

[17] Nvidia. cuSPARSE library 7.5, 2015.

[18] Nvidia. Nvidia CUDA C Programming Guide 7.5, 2015.

[19] Nvidia. Nvidia Performance Primitives 7.5, 2015.

[20] Nvidia. Nvidia Tesla P100, 2016.

[21] S. Sarkar, T. Majumder, A. Kalyanaraman, et al. Hardware accelerators for biocomputing: A survey. In *Circuits and Systems (ISCAS), Proceedings of 2010 IEEE International Symposium on*, pages 3789–3792. IEEE, 2010.

[22] T. Shimoda, S. Suzuki, M. Ohue, et al. Protein-protein docking on hardware accelerators: comparison of GPU and MIC architectures. *BMC Syst Biol*, 9(Suppl 1):S6, 2015.

[23] S. U. Thiagarajan, C. Congdon, S. Naik, et al. Intel Xeon Phi Coprocessor Developer's Quick Start Guide, 2013.
