# Supplementary material for: Graphics processing units in bioinformatics, computational biology and systems biology
Source: Brief Bioinform. 2016 Jul 7;18(5):870–85. doi: 10.1093/bib/bbw058 (PMC5862309; doi:10.1093/bib/bbw058)
Supplement: Supplementary Data [file bbw058_supplfile_3.pdf]

Supplementary File 3

List of Nvidia Video Cards Used to Test GPU-powered Tools

Marco S. Nobile, P. Cazzaniga, A. Tangherloni, D. Besozzi

Model	Architecture, Compute Capability	Peak Processing Power (GFlops in single precision)	CUDA Cores	Base Clock (MHz)	Global Memory (MB)	Power Consumption (W)	Release Date
GeForce 8800 GTX	Tesla, 1.0	345.6	128	575	768	155	November 2006
GeForce 8600 GTS	Tesla, 1.1	139.2	32	675	256	71	April 2007
Tesla C870	Tesla, 1.0	518.4	128	1350	1536	171	May 2007
GeForce 8800 GT	Tesla, 1.1	336	112	600	512	125	October 2007
GeForce 9800 GX2	Tesla, 1.1	768	256	600	1024	197	March 2008
GeForce GTX 280	Tesla, 1.3	933.12	240	602	1024	236	June 2008
Quadro FX 5800	Tesla, 1.3	622.1	240	610	4096	189	November 2008
GeForce GTX 285	Tesla, 1.3	1062.72	240	648	1024	204	January 2009
GeForce GTX 295	Tesla, 1.3	1788.4	480	576	1792	289	January 2009
GeForce GTX 480	Fermi, 2.0	1345	480	700	1536	250	March 2010
Tesla C2050	Fermi, 2.0	1030.4	448	1150	3072	238	May 2010
Tesla C2070	Fermi, 2.0	1030.4	448	1150	6144	238	September 2010
GeForce GTX 580	Fermi, 2.0	1581.1	512	772	3072	244	November 2010
GeForce GTX 570	Fermi, 2.0	1405.4	480	732	1280	219	December 2010
GeForce GTX 590	Fermi, 2.0	2488.3	1024	607	3072	365	March 2011
Tesla M2050	Fermi, 2.0	1030.4	448	575	3072	225	March 2011
Tesla M2070	Fermi, 2.0	1030.4	448	575	6144	247	March 2011
Tesla M2075	Fermi, 2.0	1030.4	448	575	6144	225	March 2011
Tesla M2090	Fermi, 2.0	1332.2	512	1300	6144	225	March 2011
GeForce GTX 680	Kepler, 3.0	2×2810.88	1536	915	4096	300	March 2012
Tesla K10	Kepler, 3.0	2288	1536	745	8192	225	May 2012
Tesla K20X	Kepler, 3.5	3520	2688	706	5120	225	November 2012
GeForce GTX Titan	Kepler, 3.5	4494	2688	837	6144	250	February 2013

Table 1: List of the Nvidia video cards exploited in the works presented in this review. It is worth noting that the increment of the processing power (expressed as billions of floating point operations per second) is mainly due to the increase of the number of available CUDA cores, while the base clock frequency is not following the same trend. We also observe an increment of the global memory size, while the power consumption is almost constant.
